# Stabilization of glucose-6-phosphate dehydrogenase oligomers enhances catalytic activity and stability of clinical variants

**DOI:** 10.1016/j.jbc.2022.101610

**Published:** 2022-01-20

**Authors:** Adriana Ann Garcia, Irimpan I. Mathews, Naoki Horikoshi, Tsutomu Matsui, Manat Kaur, Soichi Wakatsuki, Daria Mochly-Rosen

**Affiliations:** 1 Department of Chemical and Systems Biology, School of Medicine, Stanford University, Stanford, California, USA; 2 Stanford Synchrotron Radiation Lightsource, SLAC National Accelerator Laboratory, Menlo Park, California, USA; 3 Life Science Center for Survival Dynamics, University of Tsukuba, Tsukuba, Ibaraki, Japan; 4 Biological Sciences Division, SLAC National Accelerator Laboratory, Menlo Park, California, USA; 5 Department of Structural Biology, School of Medicine, Stanford University, Stanford, California, USA

**Keywords:** G6PD deficiency, therapeutic strategy, oligomer stabilization, enzymopathy, erythrocytes, dL, dimer-locked, G6P, glucose-6-phosphate, G6PD, glucose-6-phosphate dehydrogenase, G6PD^def^, G6PD deficiency, MW, molecular weight, PDB, Protein Data Bank, RBCs, red blood cells, ROS, reactive oxygen species, SAXS, small-angle X-ray scattering, SEC, size-exclusion chromatography, tL, tetramer-locked, WHO, World Health Organization

## Abstract

Glucose-6-phosphate dehydrogenase (G6PD) deficiency is a genetic trait that can cause hemolytic anemia. To date, over 150 nonsynonymous mutations have been identified in G6PD, with pathogenic mutations clustering near the dimer and/or tetramer interface and the allosteric NADP^+^-binding site. Recently, our lab identified a small molecule that activates G6PD variants by stabilizing the allosteric NADP^+^ and dimer complex, suggesting therapeutics that target these regions may improve structural defects. Here, we elucidated the connection between allosteric NADP^+^ binding, oligomerization, and pathogenicity to determine whether oligomer stabilization can be used as a therapeutic strategy for G6PD deficiency (G6PD^def^). We first solved the crystal structure for G6PD^K403Q^, a mutant that mimics the physiological acetylation of wild-type G6PD in erythrocytes and demonstrated that loss of allosteric NADP^+^ binding induces conformational changes in the dimer. These structural changes prevent tetramerization, are unique to Class I variants (the most severe form of G6PD^def^), and cause the deactivation and destabilization of G6PD. We also introduced nonnative cysteines at the oligomer interfaces and found that the tetramer complex is more catalytically active and stable than the dimer. Furthermore, stabilizing the dimer and tetramer improved protein stability in clinical variants, regardless of clinical classification, with tetramerization also improving the activity of G6PD^K403Q^ and Class I variants. These findings were validated using enzyme activity and thermostability assays, analytical size-exclusion chromatography (SEC), and SEC coupled with small-angle X-ray scattering (SEC-SAXS). Taken together, our findings suggest a potential therapeutic strategy for G6PD^def^ and provide a foundation for future drug discovery efforts.

Glucose-6-phosphate dehydrogenase (G6PD) is a housekeeping enzyme that generates NADPH. The NADPH produced by G6PD provides the cell with reductive power and is essential for the regeneration of oxidized glutathione—reduced NADPH levels cause redox imbalance (1). Due to G6PDs essential role in cell metabolism, other enzymes can compensate for loss of G6PD function. However, G6PD is the primary source of NADPH in erythrocytes or red blood cells (RBCs). Consequently, loss of G6PD function renders erythrocytes vulnerable to reactive oxygen species (ROS) and can cause hemolytic anemia.

G6PD deficiency (G6PD^def^) is a genetic trait present in ∼400 million people worldwide (2). To date, over 150 unique nonsynonymous mutations have been identified in the G6PD gene (3). Because each mutation is unique, they can have different effects on G6PD function and can cause varying severities of hemolytic anemia, and the World Health Organization (WHO) established a classification system for G6PD^def^. Classifications are based on clinical outcome and residual G6PD activity in RBCs: Class I < 10% G6PD activity with chronic nonspherocytic hemolytic anemia, Class II < 10% G6PD activity with intermittent hemolytic anemia, Class III 10 to 60% G6PD activity with hemolytic anemia after exposure to oxidative stress, and Class IV > 60% G6PD activity and are asymptomatic (4, 5). Although Class I variants are the most severe, they are relatively rare and are often reported in single clinical case studies. Their low allele frequency is likely the result of G6PDs essential role in development; knockdown of G6PD in mice is embryonic lethal (6). In contrast, Class II and III variants are more common; however, they are generally asymptomatic when following strict dietary and drug restrictions (7). Currently, blood transfusions remain the only treatment for an acute hemolytic event, and there are no therapeutics for the treatment of G6PD^def^ (8).

G6PD exists as a monomer, homodimer, and homotetramer, with each monomer having three binding sites: one for catalytic glucose-6-phosphate (G6P), one for an NADP^+^ cofactor, and an allosteric NADP^+^-binding site. The latter allosteric NADP^+^-binding site, also known as the structural NADP^+^-binding site, is important for maintaining long-term protein stability and for promoting oligomer assembly (9, 10, 11, 12). Oligomerization is central to G6PD function as it controls the activity of G6PD; the monomer is inactive with both the dimer and tetramer being the active forms. These properties are essential for normal G6PD function and loss of structural NADP^+^ binding and oligomerization cause pathogenicity: Class I variants cluster at the dimer interface and the structural NADP^+^-binding site, and Class II variants cluster at the tetramer interface (13, 14).

Despite oligomerization being critical for G6PD activity, it has remained unclear if there are catalytic differences between dimeric and tetrameric G6PD since activity assays utilize NADP^+^ and G6P, both of which alter the oligomeric state of G6PD (15). Recent evidence suggests that there are catalytic differences between the dimer and tetramer, as locking G6PD in the tetrameric state allowed for the first direct biochemical comparison; tetrameric G6PD had improved catalytic efficiency over dimeric G6PD (16). To our knowledge, the oligomeric state is rarely assessed in patient samples. The only known study to do so was by Mizukawa *et al.* (17) they performed native PAGE on human red blood cell lysate and found that the predominant species for wild-type G6PD, G6PD^WT^, are tetramers, followed by dimers, and then monomers. Furthermore, they showed that homo-oligomerization was significantly reduced in patient samples harboring clinical mutations.

Our lab identified a small-molecule activator (AG1) of G6PD, which improves the activity and stability of several Class II and III G6PD clinical variants *via* stabilization of structural NADP^+^ and the dimer (18, 19). These findings suggest that stabilization of oligomerization or NADP^+^ binding can restore enzyme function. Here, we investigate loss of structural NADP^+^ binding, its contribution to oligomerization and pathogenic outcome, and whether stabilization of distinct oligomeric forms can be used as therapeutic strategy for treating G6PD^def^.

## Results

### Loss of structural NADP^+^ binding disrupts substrate binding and tetramerization

**Figure 1:**
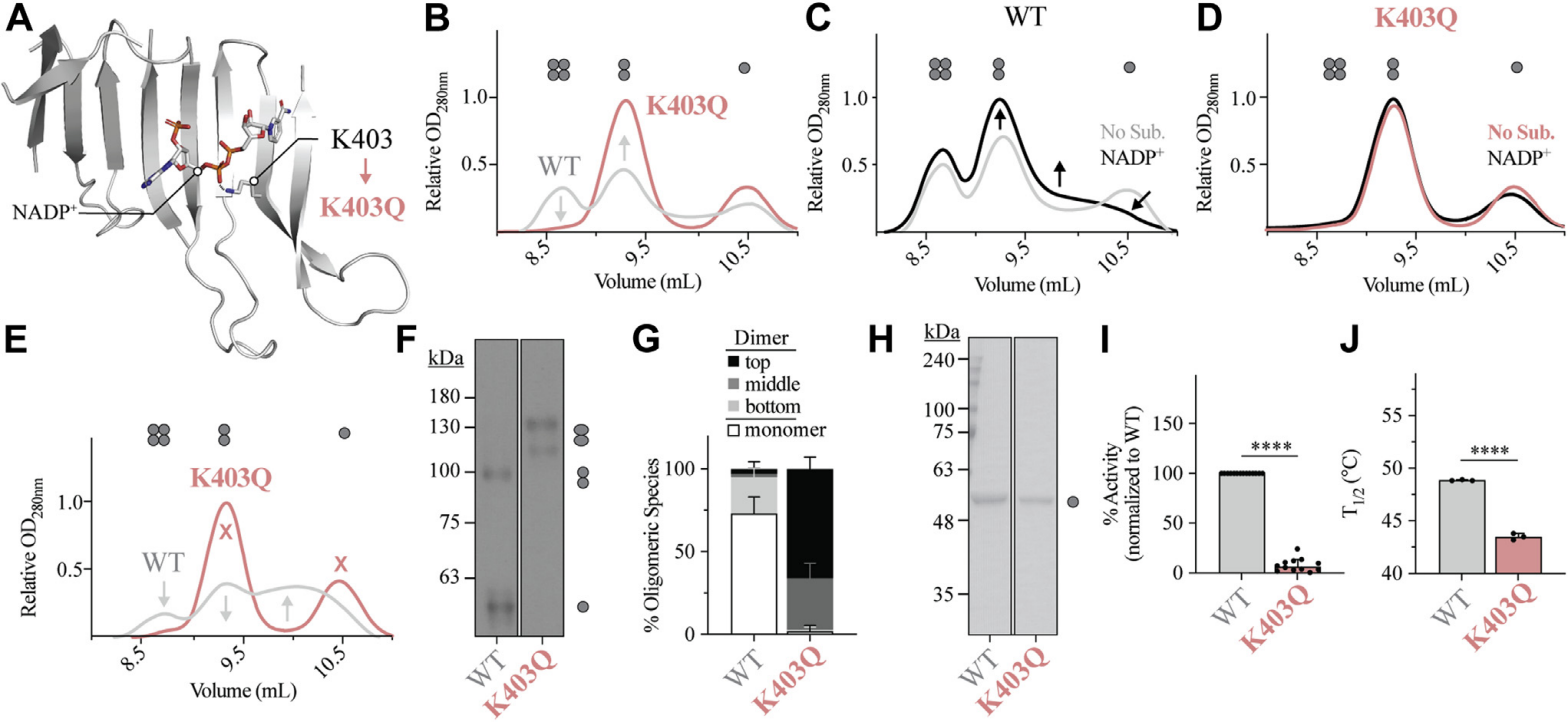
**Loss of structural NADP**^**+**^**binding affects G6PD oligomerization, activity, and stability.***A*, location of the K403 residue on the ß-sheet of the structural NADP^+^-binding site and its interaction with the NADP^+^ phosphate group in G6PD^WT^ (PDB 6E08). *B*, the SEC chromatogram for G6PD^WT^ and G6PD^K403Q^ in the absence of NADP^+^. *C* and *D*, the SEC chromatogram for G6PD^WT^ and G6PD^K403Q^ in the absence or presence of 10 μM NADP^+^ (for *B*–*D*, *n* = 1 for each condition). *E*, the SEC chromatogram in the absence of NADP^+^, changing pH, ionic strength, and adding 10 mM G6P (*n* = ≥2 for each mutant). *F* and *G*, P-Native PAGE Western blot and quantification (*n* ≥ 3 for each mutant). *H*, Coomassie of SDS-PAGE (*n* = 2 for each mutant), (*I*) activity (*n* = 14 for each mutant), and (*J*) thermostability for G6PD^WT^ and G6PD^K403Q^ (*n* = 3 for each mutant). For P-Native PAGE, differences between G6PD^K403Q^ and G6PD^WT^ were detected for each group of oligomeric species *via* four two-tailed unpaired *t test*s (*p* value ≤ 0.0004 for each *t* test). Full Western blot and Coomassie images for *F* and *H* are provided in Figs. S10 and S11. G6PD, glucose-6-phosphate dehydrogenase.

Structurally, lysine 403 (K403), forms an important contact with a phosphate group of the structural NADP^+^ (Fig. 1*A*), and studies by Wang *et al.* (12) revealed that K403 is acetylated in RBCs under physiological conditions. Mutation of K403 to glutamine (K403Q) mimics acetylation, with both acetylation and K403Q disrupting oligomerization and G6PD activity in cells. To understand how loss of structural NADP^+^ binding affects G6PD oligomerization in both a pathological and a physiological context, we generated G6PD^K403Q^ for *in vitro* characterization.

To assess the oligomeric state of G6PD, analytical size-exclusion chromatography (SEC) was used, and a buffer condition where monomeric, dimeric, and tetrameric species could be observed for G6PD^WT^ was selected: 500 nM G6PD, 80 mM sodium phosphate (NaP) (pH 8.0). The enzymes used in subsequent assays were expressed and purified as indicated in the Experimental procedures section. In the presence of 80 mM NaP (pH 8.0), G6PD^WT^ consisted of approximately 25% tetramer, 40% dimer, and 22% monomer, with each species clearly defined (Figs. 1*B* and S1*A*, % numbers expressed in monomer equivalent throughout this manuscript). Under the same condition, no G6PD^K403Q^ tetramer was observed (Figs. 1*B* and S1*B*). Instead, 74% of G6PD^K403Q^ existed as a dimer, with no change in the percentage of monomer. Interestingly, an intermediate species was detected between the dimer and monomer peaks of G6PD^WT^; however, the intermediate was not detected for G6PD^K403Q^. This intermediate is likely the result of a dynamic dimer–monomer equilibrium, with intermediate SEC species reported for G6PD (20).

When incubating G6PD^WT^ with 10 μM NADP^+^, NADP^+^ did not alter the relative percentage of tetramer; however, it slightly increased the dimer (∼8%), resulting in a subsequent reduction of the monomer (Figs. 1*C* and S1*C*). Additionally, the monomer migrated through the column faster, as indicated by a slight shift in the monomer elution volume, from 10.5 ml to 10.25 ml. In contrast, NADP^+^ had no effect on G6PD^K403Q^ oligomerization (Figs. 1*D* and S1*D*); there were no changes in the distribution of species and no changes in location of peaks, which is consistent with the inability of G6PD^K403Q^ to bind structural NADP^+^.

When incubating G6PD^WT^ and G6PD^K403Q^ in 50 mM Tris (pH 7.4), 150 mM NaCl, and 10 mM G6P, conditions that should dissociate the dimer (10), G6PD^WT^ oligomerization was severely impacted; the percentages of tetramers and dimers were both reduced, while the percentages of intermediate species and monomers increased (Figs. 1*E* and S1*E*). In contrast, G6PD^K403Q^ had minor changes in the relative distribution of oligomers (Figs. 1*E* and S1*F*). Since differences in G6P binding may explain why G6PD^WT^ oligomerization was severely impacted but not G6PD^K403Q^, we removed G6P. In the absence of G6P, we still observed major differences in the G6PD^WT^ oligomeric distribution; a 16% reduction in the tetramer, a 15% reduction in dimer, and a 34% increase in monomer (Fig. S1*G*). Therefore, changes in the buffer composition alone affect the quaternary structure for G6PD^WT^, but not for G6PD^K403Q^. The fact that G6PD^WT^ had an increase in the relative percentage of monomers but G6PD^K403Q^ did not suggests that the G6PD^K403Q^ dimer has a higher binding affinity than G6PD^WT^ in this condition, and loss of structural NADP^+^ binding favors the dimeric state.

With the G6PD^K403Q^ dimer less prone to dissociation, it should have a higher propensity to form a tetramer; however, lack of tetramer formation suggests that although the dimer is present, loss of structural NADP^+^ binding may induce allosteric changes that prevent the tetramer from forming. Using partial native PAGE (P-Native PAGE), described in previous work (18), differences between the G6PD^WT^ and G6PD^K403Q^ dimer were observed. Consistent with our SEC data, G6PD^WT^ was more prone to monomer–monomer dissociation, as observed by the presence of more monomeric G6PD (Fig. 1, *F* and *G*). However, unlike SEC, three bands were observed near the expected molecular weight (MW) of the G6PD dimer (116 kDa), and major differences between G6PD^WT^ and G6PD^K403Q^ were observed; the G6PD^K403Q^ dimer had a higher apparent MW than the G6PD^WT^ dimer.

To validate differences observed on P-Native PAGE were due to inherent differences in the dimer, G6PD^WT^ and G6PD^K403Q^ were subjected to SDS-PAGE, and no differences between the monomer MW were detected, confirming that MW differences were due to differences in mobility (Fig. 1*H*). Interestingly, although G6PD^K403Q^ forms a dimer that is less prone to dissociation, it is inactive and thermally unstable (Fig. 1, *I* and *J*). Loss of activity can be explained by loss of catalytic NADP^+^ and/or G6P binding as the Michalis–Menten equation could not reproducibly fit our data (K_m_ > 1 mM) with maximum substrate concentrations reaching 1 mM G6P and NADP^+^. Given that activity, thermostability, and amino acid location correlate with the clinical severity of G6PD variants (13), G6PD^K403Q^ likely mimics a Class I variant—low activity, low thermostability, and located at the structural NADP^+^-binding site.

### Class I variants located at and distant from the structural NADP^+^-binding site mimic G6PD^K403Q^

**Figure 2:**
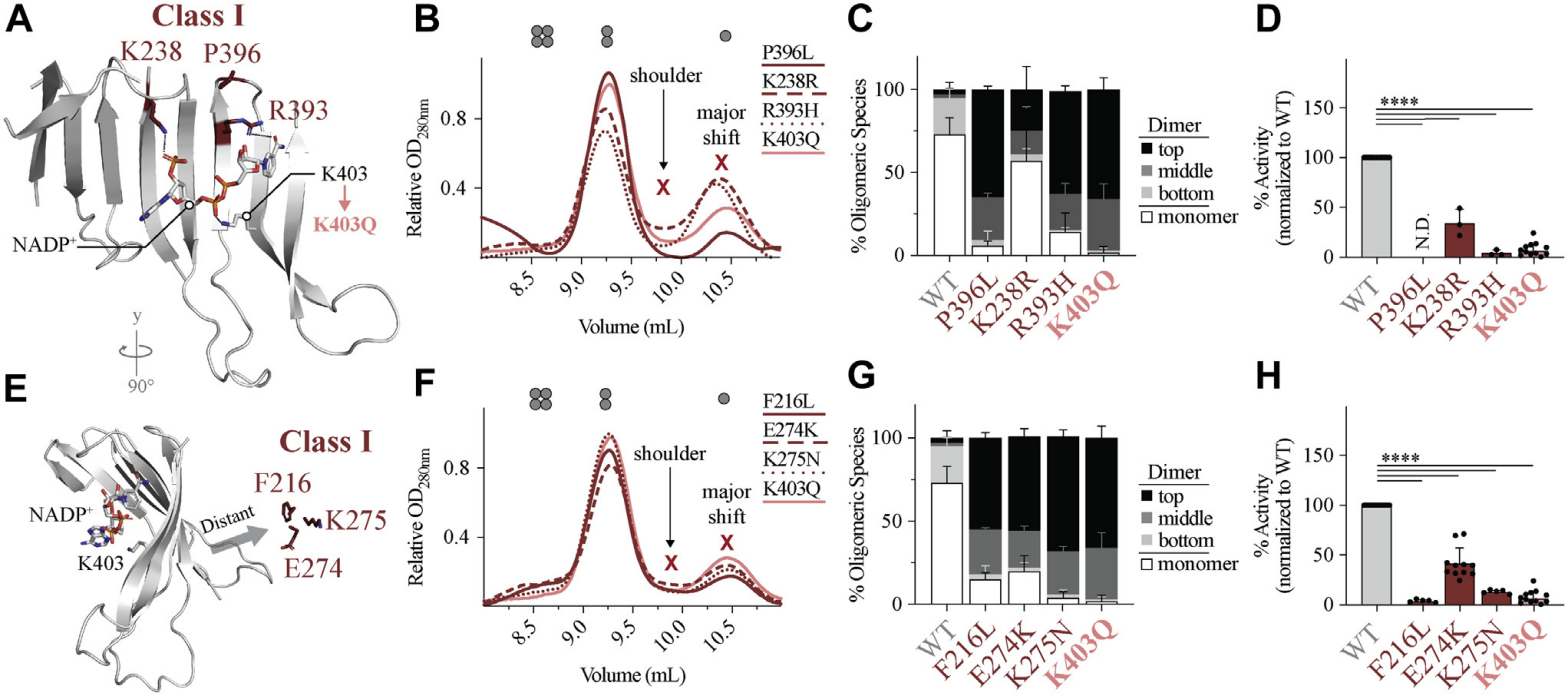
**The impact of loss of structural NADP**^**+**^**binding on G6PD oligomerization, activity, and stability in Class I variants at and distant from the structural NADP**^**+**^**-binding site.***A*, location of Class I mutations at the structural NADP^+^-binding site and their interactions with the structural NADP^+^ in G6PD^WT^ (PDB 6E08). For Class I mutations located at the structural NADP^+^-binding site, (*B*) the SEC chromatogram (*n* = 2 for each mutant), (*C*) quantification of P-Native PAGE (*n* ≥ 3 for each mutant), (*D*) and activity (*n* ≥ 3 for each mutant). *E*, location of Class I mutations distant from the ß-sheet of the structural NADP^+^-binding site in G6PD^WT^ (PDB 6E08). For Class I mutations distant from the structural NADP^+^-binding site, (*F*) the SEC chromatogram (*n* = 2 for each mutant), (*G*) P-Native PAGE quantification (*n* ≥ 3 for each mutant), (*H*) and activity (*n* ≥ 3 for each mutant). For P-Native PAGE, differences between mutant and G6PD^WT^ were detected for each group of oligomeric species *via* four one-way ANOVAs (*p* value < 0.05 for each ANOVA). G6PD, glucose-6-phosphate dehydrogenase; SEC size-exclusion chromatography.

Like G6PD^K403Q^, multiple Class I pathogenic mutations exist near the structural NADP^+^-binding site. To confirm that G6PD^K403Q^ is representative of a Class I mutant, three clinical Class I variants were examined (Fig. 2*A*): G6PD^P396L^, G6PD^R393H^, and G6PD^K238R^, with G6PD^K238^ and G6PD^R393H^ forming direct contacts with the structural NADP^+^. Like G6PD^K403Q^, Class I variants at the structural NADP^+^-binding site have impaired NADP^+^ binding (Fig. 2*B*); the monomer peak of the SEC chromatogram displayed a well-defined peak at 10.5 ml in the presence of 10 μM NADP^+^. Additionally, the dimer was less prone to dissociation, formed a dimer, which migrates slower on P-Native PAGE, could not form a tetramer, and had reduced catalytic activity (Fig. 2, *C* and *D*).

Interestingly, for G6PD^K238R^, although the activity is compromised, it maintained 34% activity, whereas G6PD^P396L^, G6PD^R393H^, and G6PD^K403Q^ were catalytically inactive. Additionally, the G6PD^K238R^ dimer dissociated to a greater extent and formed less of the top dimer species. G6PD^K238R^ may retain activity due to the nature of the amino acid substitution; lysine and arginine have a similar size and positive charge. This may reduce, but not eliminate, structural NADP^+^ binding.

To understand if other Class I mutations, where the mutation is distant from the structural NADP^+^-binding site, exhibit the same features, three additional Class I mutations, G6PD^F216L^, G6PD^E274K^, G6PD^K275N^, distant from the structural NADP^+^-binding site were generated (Fig. 2*E*). Like mutations located at the structural NADP^+^-binding site, G6PD^F216L^, G6PD^E274K^, G6PD^K275N^ displayed properties like G6PD^K403Q^ (Fig. 2, *F*–*H*). This suggests that mutations at the dimer and/or tetramer interface, with G6PD^F216L^ connecting to the dimer interface while G6PD^E274K^ and G6PD^K275N^ are located at the tetramer interface, can induce allosteric changes, which cause loss of structural NADP^+^ binding and formation of a dysfunctional dimer. It is likely all variants that exhibit these allosteric changes result in the most severe G6PD^def^.

### Structure of G6PD^K403Q^ reveals common defects between G6PD^K403Q^ and Class I mutants

**Figure 3:**
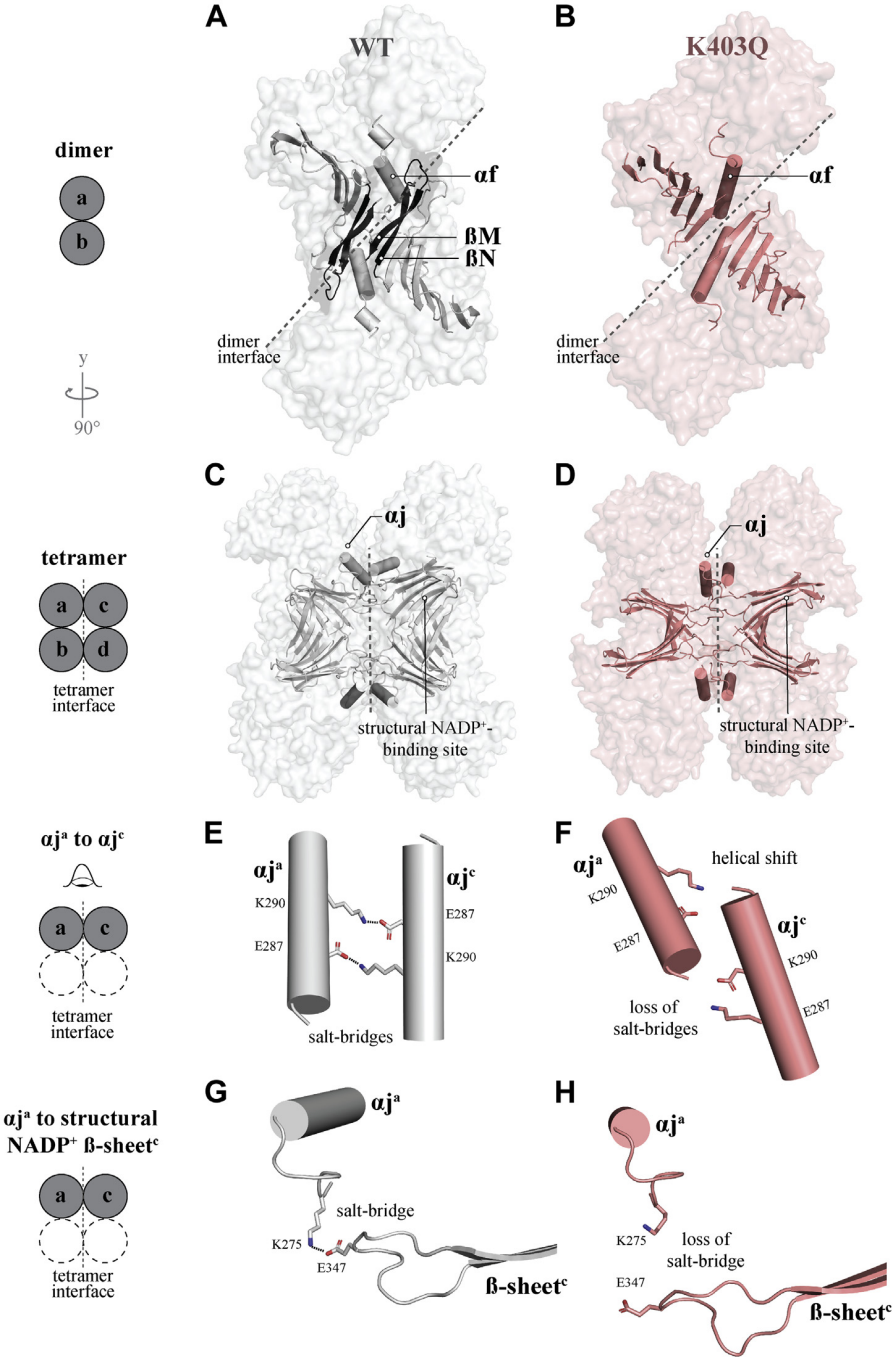
**Comparison of the G6PD**^**WT**^**and G6PD**^**K403Q**^**structures.***A* and *B*, dimeric G6PD^WT^ (PDB 6E08, *gray*) and G6PD^K403Q^ (PDB 7SEI, *salmon*) surface, with the structural NADP^+^ ß-sheet and αf-helix displayed as *cartoons*. The G6PD^K403Q^ structural NADP^+^ ß-sheet is missing two ß-strands M and N, which are colored *black* in the G6PD^WT^ structure. *C*, the G6PD^WT^ (PDB 6E08) tetramer, with the αj-helix and structural NADP^+^-binding site displayed as cartoons. *D*, the G6PD^K403Q^ (PDB 7SEI) assembled tetramer, with the αj-helix and structural NADP^+^-binding site displayed as cartoons. *E*, the αj-helix forms critical salt-bridge interactions with its symmetry mate across the tetramer interface (*F*), and these interactions are lost in G6PD^K403Q^. *G*, K275 is located downstream from the αj-helix and forms a salt bridge with E347 across the tetramer interface in G6PD^WT^, connecting the structural NADP^+^ ß-sheet to the αj-helix. *H*, this interaction is lost in G6PD^K403Q^.

To confirm whether the altered biochemical and biophysical properties of G6PD^K403Q^ were the result of changes in protein structure, we solved the crystal structure of G6PD^K403Q^ (PDB 7SEI) at 3.65-Å resolution and observed significant structural changes (Figs. 3 and S2). Firstly, and consistent with our hypothesis, the mutation of lysine to glutamine disrupted structural NADP^+^ binding—no density was observed for structural NADP^+^. Additionally, no density was observed for the ßM and ßN strands of the structural NADP^+^-binding site (shown in black on G6PD^WT^ in Fig. 3*A*), which caused major changes in the globular structure of the dimer (Fig. 3, *A* and *B* and Movies S1 and S2, white to salmon). Secondly, major alterations were observed in the αf-helix; the αf-helix was extended, causing a loop to protrude into the G6P-binding pocket (Fig. S2*C* and Movie S3). Interestingly, electron density for the catalytic NADP^+^ was observed (Fig. S2, *A* and *B*); though, loss of G6P binding inhibits its conversion.

These findings are consistent with recently solved structures of several G6PD Class I variants (21), suggesting that Class I variants and posttranslational modifications located at the structural NADP^+^-binding site share a similar structural defect and that a dysfunctional dimer may be a means to regulate G6PD^WT^ activity. It should be noted that the resolution of G6PD^K403Q^ is relatively low, 3.65-Å, with the crystallographic structure having high B-factors. However, we have previously characterized five other Class I and Class I-like dimer structures of G6PD, all having mid to poor resolution with high B-factors, with 6VAQ having the highest resolution—2.95-Å. All of these structures share the same dimer and structural features, which are distinct from the G6PD^WT^ dimer.

To understand how structural changes in the dimer impact tetramerization, a hypothetical G6PD^K403Q^ tetramer was generated by reflecting a biological G6PD^K403Q^ dimer along the axis of symmetry, and the G6PD^K403Q^ tetramer was compared with the G6PD^WT^ tetramer (Fig. 3, *C* and *D* and Movie S4). Normally, the G6PD^WT^ tetramer interface is stabilized by several salt-bridge interactions: (1) salt bridges between K290 of one monomer and E287 of another monomer stabilize opposing αj-helices across the tetramer interface (Fig. 3*E*). (2) K275, a residue extending from the αj-helix, forms a salt-bridge with E347, which is located on a ß-sheet loop extending across the tetramer interface on an opposing monomer (Fig. 3*G*). However, in G6PD^K403Q^, the αj-helix is shifted, preventing K290/E287 and E347/K275 salt bridges from forming, explaining loss of tetramerization (Fig. 3, *F* and *H* and Movies S5 and S6).

### The G6PD^WT^ tetramer is more active and stable than the G6PD^WT^ dimer

**Figure 4:**
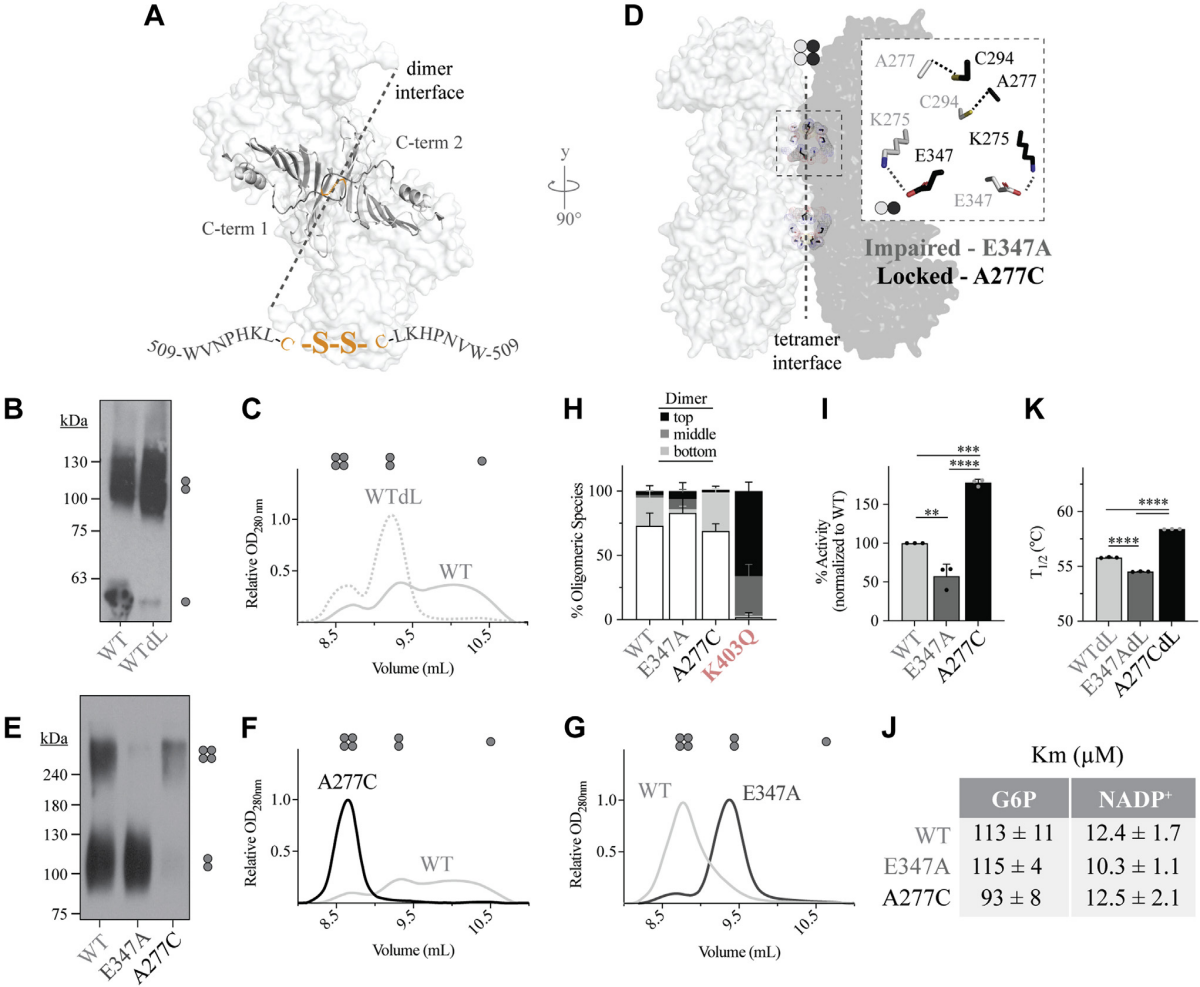
**Generation and characterization of synthetic G6PD mutants, either locked in the dimer or tetramer state or tetramer inhibited.** Mutagenic strategy is as follows: *A*, for the dimer-locked (dL) mutant, a cysteine was added to the C-terminal tail; the C-terminal tails align at the dimer interface (PDB 2BH9). *B* and *C*, P-Native PAGE after crosslinking with 1% glutaraldehyde and SEC chromatogram for G6PD^WT^ and G6PD^WTdL^ mutant (*n* = 1, *n* ≥ 2 for each mutant respectively). *D*, for the tetramer-locked (tL) mutant, a A277 was mutated to a cysteine, which is near C294 at the tetramer interface. Additionally, a salt bridge at the tetramer interface was disrupted to generate the tetramer impaired mutant, G6PD^E347A^ (PDB 6E08). *E*, P-Native PAGE Western blot after cross-linking with 1% glutaraldehyde (*n* = 3 for each mutant). *F*, the SEC chromatogram in a condition that dissociates the G6PD^WT^ dimer and (*G*) a condition that promotes G6PD^WT^ tetramerization (*n* ≥ 2 for each mutant). *H*, P-Native PAGE Western blot quantification for synthetic variants (*n* ≥ 3 for each mutant). Biochemical analysis of synthetic variants including (*I*) activity (*n* = 3 for each mutant), (*J*) K_m_ for G6P and NADP^+^ (*n* = 3 for each mutant), and (*K*) thermostability (*n* = 3 for each mutant). For P-Native PAGE, differences between mutant and G6PD^WT^ were detected for each group of oligomeric species *via* four one-way ANOVAs (*p* value < 0.05 for each ANOVA). Full Western blot images for *B* and *E* are provided in Fig. S11. G6PD, glucose-6-phosphate dehydrogenase; SEC size-exclusion chromatography.

Since G6PD^K403Q^ and Class I variants could not form a tetramer, we aimed to understand how the tetramer contributes to enzymatic activity and stability. To do this, we made synthetic mutants favoring distinct oligomeric states. Nonnative cysteines were introduced at either the dimer or tetramer interface to cross-link G6PD oligomers together. For generation of the dimer-locked (dL) synthetic mutant, a cysteine was added to the C-terminal end; the C-terminal tail of monomer-1 faces the C-terminal tail of monomer-2 at the dimer interface (Fig. 4*A*). In theory, a disulfide bond would bridge monomer-1 to monomer-2. Indeed, this was the case as the G6PD^WTdL^ monomer was not present on P-Native PAGE following cross-linking and did not dissociate to a monomer on SEC in monomer conditions (Fig. 4, *B* and *C*).

To generate synthetic mutants stabilized in or inhibited from being in the tetrameric state, we followed work done by Ranzani *et al.* (16); we mutated an alanine to a cysteine (G6PD^A277C^)—to form a disulfide bond across the tetramer interface, and we disrupted a salt bridge between E347 and K275 across the tetramer interface (G6PD^E347A^)—to prevent tetramerization (Fig. 4*D*). These mutations are effective due to each disulfide bond pair being multiplied by 4 in the tetramer interface. Consistent with findings by Ranzani *et al.*, the G6PD^A277C^ mutant forms a stable tetramer that does not dissociate when subjected to monomer conditions (Fig. 4, *E* and *F*). Additionally, the G6PD^E347A^ mutant does not form a tetramer when placed in conditions favoring tetramer formation; low pH, ionic strength, with addition of NADP^+^ and MgCl_2_ (Fig. 4, *E* and *G*). These mutations did not modify the biophysical properties of the dimer, as G6PD^E347A^ and G6PD^A277C^ behaved similarly to G6PD^WT^; P-Native PAGE results show that ∼70% of G6PD^WT^ and G6PD^A277C^ was in monomeric form (Fig. 4*H*). Interestingly, G6PD^E347A^ had a slight increase in dimer dissociation, ∼80% in the monomeric form, suggesting that the tetramer may aid in preventing monomer–monomer dissociation.

When examining the enzyme kinetics of the generated mutants, we found that impairment of the tetramer, G6PD^E347A^, reduced enzymatic activity by ∼40% (Fig. 4*I*). Consistent with these findings, stabilization of the tetramer, G6PD^A277C^, increased the catalytic activity by ∼80%, reinforcing the importance of tetrameric G6PD for catalysis. When comparing the dimeric form, G6PD^E347A^, against the tetrameric form, G6PD^A277C^, the tetramer had ∼300% increase in catalytic activity. The K_m_ for NADP^+^ and G6P for G6PD^WT^, G6PD^E347A^, and G6PD^A277C^ supports increased activity as the tetramer binds G6P more readily; a K_M_ of 115 *versus* 93 μM for G6PD^E347A^ and G6PD^A277C^, respectively (Fig. 4*J*). Additionally, the tetramer is more thermally stable than dimeric G6PD (Fig. 4*K*). Together, these findings suggest that the tetrameric state provides maximum enzymatic activity and stability of G6PD.

### Locking G6PD^K403Q^ in the tetrameric state improves activity and stability

Previous work in the lab found that stabilizing the dimeric state with a small molecule increases the catalytic activity of Class II and III variants (18, 19). As G6PD^K403Q^ has been established as a good model for Class I variants, we sought to determine if stabilizing the dimer or tetramer could correct activity or stability for G6PD^K403Q^. Using the same mutagenic strategy described above, we made double and triple mutants, the G6PD^K403Q^ mutation combined with either dL, or both dL and tetramer-locked (tL), to determine if inducing oligomerization could recover activity and/or stability for G6PD^K403Q^.

**Figure 5:**
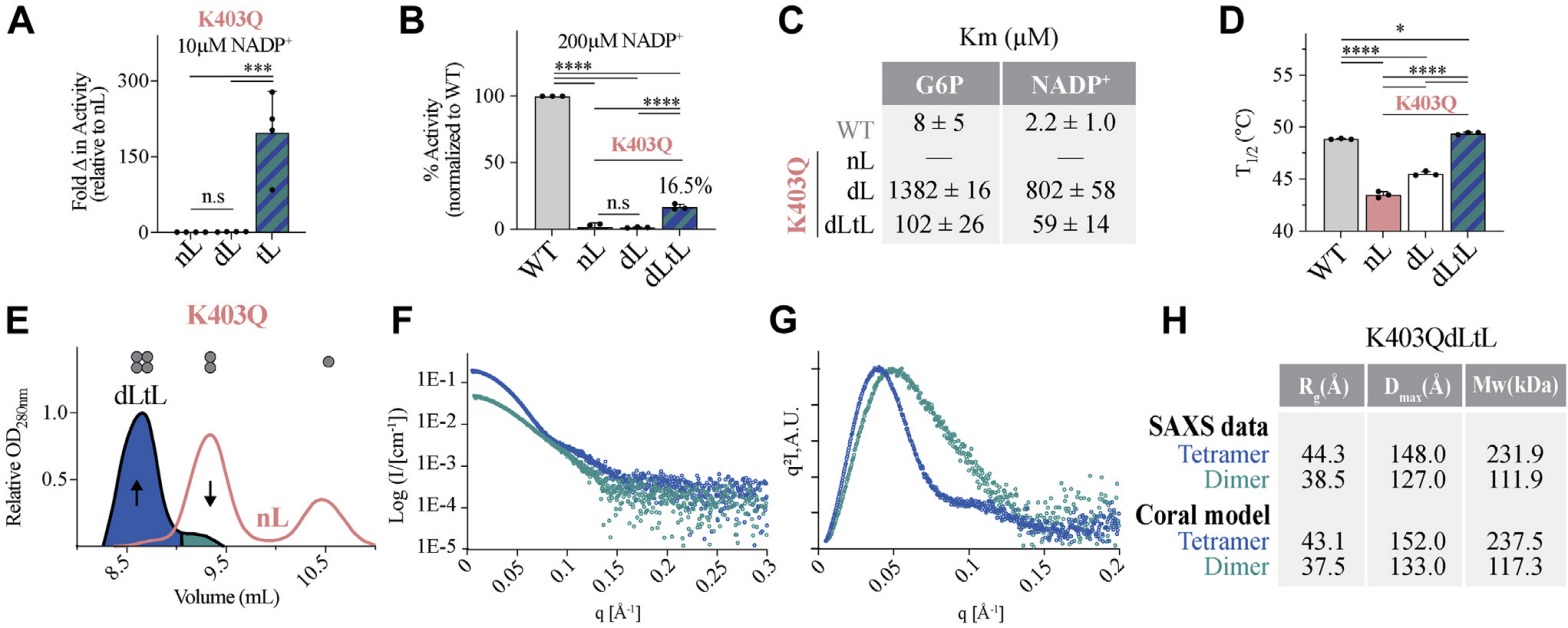
**The biophysical and biochemical characterization of G6PD**^**K403Q**^**double and triple mutants locked in the dimer and/or tetrameric state.** Activity of G6PD^K403Q^ mutants at (*A*) 10 μM (*n* = 4 for each mutant) and (*B*) 200 μM NADP^+^ displayed relative to G6PD^WT^ (*n* = 3 for each mutant). *C*, the K_m_ values for G6PD^WT^ and G6PD^K403Q^ mutants (*n* = 3 for each mutant). *D*, the thermostability of G6PD^WT^ and G6PD^K403Q^ mutants (*n* = 3 for each mutant). *E*, the SEC chromatogram of G6PD^K403Q^ and G6PD^K403QdLtL^ in the presence of 10 mM G6P (*n* ≥ 2 for each mutant). SEC-SAXS data for K403QdLtL dimer and tetramer fractions: (*F*) the I(q) *versus* q as a log-linear plot, (*G*) the Kratky plot, and (*H*) the MW estimations from experimental SAXS data and CORAL modeling. MW, molecular weight; SAXS, small-angle X-ray scattering.

Consistent with our findings that G6PD^K403Q^ forms a stable dimer, locking G6PD^K403Q^ in the dimeric states offered little improvement in activity (Fig. 5*A*). In contrast, locking G6PD^K403Q^ in the tetrameric state improved activity significantly, ∼200-fold. Similar trends were obtained at substrate concentrations recommended by the WHO (200 μM NADP^+^ and 600 μM G6P) and when comparing with G6PD^WT^, tetramer stabilization activated a catalytically dead enzyme to 16.5% of G6PD^WT^ activity (Fig. 5*B*).

To better understand the relationship between substrates and activity, the Michalis–Menten kinetics were measured for the G6PD^K403Q^ series (Fig. 5*C*). The K_m_ reveals that G6PD^K403QdL^ improved both G6P and NADP^+^ binding, as we were able to generate a reliable fit to the Michalis– Menten equation, whereas G6PD^K403Q^ could not (previously discussed). However, although dimer locking improved the K_m_ for G6P and NADP^+^, the K_m_ exceeded 800 μM, binding substrates poorly. Consistent with the fact that tetramer locking was able to activate G6PD^K403Q^, G6PD^K403QdLtL^ improved G6P and NADP^+^ binding significantly, as the K_m_ changed from 1382 to 102 μM for G6P and from 802 to 59 μM for NADP^+^. The K_m_ for G6PD^WT^ appears different (Figs. 4*J* and 5*C*) because different protein concentrations were used; ∼3 nM was used for Figure 4*J*, whereas ∼70 nM was used for Figure 5*C*—higher concentrations were needed to accurately measure G6PD^K403Q^ kinetics.

The physiological concentration of NADP^+^ in erythrocytes has been reported at 25 to 40 μM NADP^+^ (21, 22), with G6PD^def^ erythrocytes having higher concentrations of NADP^+^, 40 to 60 μM (22). Additionally, the physiological concentration of G6P has been reported at 25 to 45 μM (23, 24). Therefore, G6PD^K403QdLtL^ causes the K_m_ to fall within physiologically relevant substrate concentrations. When examining protein stability, both dimer and tetramer locking improved G6PD^K403Q^ stability; dimer locking increased protein stability by 2.0 °C with tetramer locking further increasing stability by 3.9 °C, bringing G6PD^K403QdLtL^ thermostability to that of G6PD^WT^ (Fig. 5*D*). These findings are consistent with those obtained with the impaired tetramer mutant, G6PD^E347A^, and tL mutant, G6PD^A277C^; the tetramer is more stable than the dimer.

To further confirm the biochemical and thermostability measurements, we used analytical SEC and SEC coupled with small-angle X-ray scattering (SEC-SAXS) to see if the G6PD^K403QdLtL^ indeed forms a tetramer. When running G6PD^K403Q^ and G6PD^K403QdLtL^ on SEC in monomer conditions, up to 80% of G6PD^K403QdLtL^ was a tetramer, confirming that the G6PD^K403QdLtL^ mutant can form a tetramer (Figs. 5*E* and S3*A*). To further characterize the G6PD^K403QdLtL^ structure in solution, we used SEC-SAXS, with the G6PD^K403QdLtL^ dimer and tetramer fractions having an equal distribution (Figs. S4*A* and S5*A*).

Analysis of SEC-SAXS data reveals two distinct signatures for the G6PD^K403QdLtL^ dimer and tetramer fractions, as shown in the I(q) *versus* q as a log-linear plot and the Kratky plot (Fig. 5, *F* and *G*). Additionally, *Guinier* analysis, *P*(r) analysis, and Porod volume estimates for dimer and tetramer fractions yielded different R_g_, D_max_, and MW values, corresponding to dimers and tetramers (Figs. 5*H*, S4 and S5, and Table S2). Interestingly, the Kratky plot is shifted to the right for the G6PD^K403QdLtL^ dimer (Fig. 5*G*). This indicates that the G6PD^K403QdLtL^ dimer has a greater population of flexible regions. Therefore, by forming the tetramer, the structural flexibility of the dimeric enzyme is reduced; this is consistent with and likely explains increased stability by tetramer stabilization.

We next attempted to solve the crystal structure of G6PD^K403QdLtL^ tetramer; unfortunately, we were only successful at solving the structure of G6PD^K403QdLtL^ (PDB 7SEH) in the dimeric form at 2.8 Å resolution (Fig. S8). Interestingly, the G6PD^K403QdLtL^ dimer recovered the missing ßM strand of the structural NADP^+^ ß-sheet, lost in G6PD^K403Q^ (Fig. S8*A*). Additionally, the globular shape of the dimer is modified, with a hinge-like shift in one of the monomers relative to the other monomer in the dimer (Movies S1 and S2, salmon to teal). This shift is illustrated using G341 as a reference point; there is a 39.6 Å and 55.3 Å distance between monomers of G6PD^K403Q^ and G6PD^K403QdLtL^, respectively, and the catalytic NADP^+^ site is shifted by 20.4 Å (Fig. S8*B*). These observations confirm that the dimer-locked and tetramer-locked mutations induce structural changes in the G6PD^K403QdLtL^ dimer, though the structural modifications that occur in the G6PD^K403QdLtL^ tetramer remain to be determined.

We used CORAL (25) to model the G6PD^WT^ tetramer (PDB 6E08) and G6PD^K403QdLtL^ dimer (PDB 7SEH) structures with missing flexible regions (Figs. S6 and S7, *C*–*F*), and we used these models to generate theoretical I(q) *versus* q as a log-linear plot and Kratky plots (Figs. S6 and S7, *A* and *B*). The theoretical G6PD^WT^ tetramer and G6PD^K403QdLtL^ dimer plots fit well to the experimental SAXS data for the tetramer (Fig. S6, *A* and *B*) and dimer fraction, respectively (Fig. S7, *A* and *B*). This confirms that the G6PD^K403QdLtL^ dimer crystallographic structure matches the structure in solution. Additionally, the experimental values derived from SEC-SAXS analysis agree with R_g_ (Å), D_max_ (Å), and MW estimations generated from CORAL modeling (Fig. 5*H*), further confirming that the G6PD^K403QdLtL^ mutant forms tetramer in solution.

### Locking pathogenic variants in tetrameric state improves activity for Class I variants and stability for clinical variants of different classes

**Figure 6:**
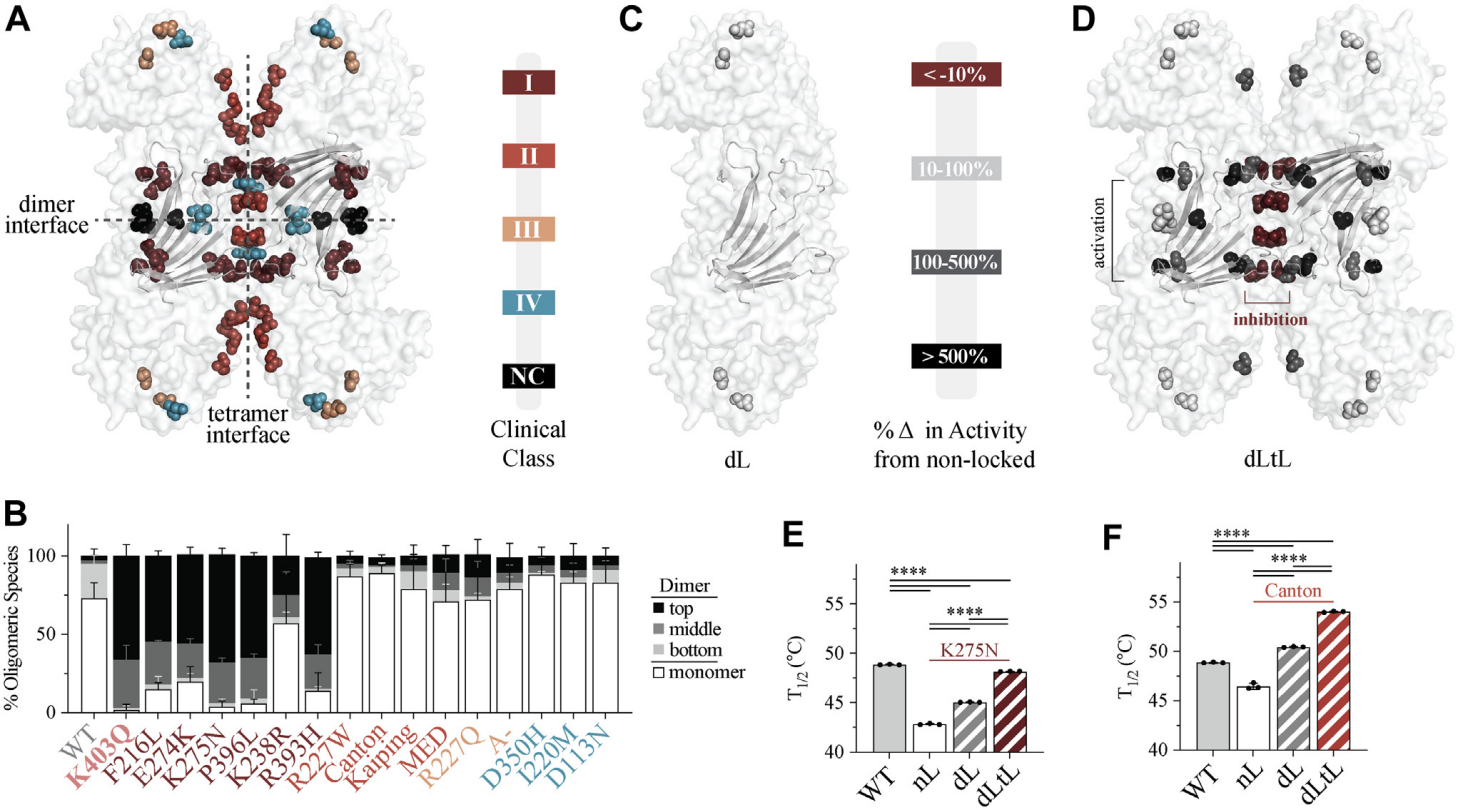
**Biochemical characterization of clinical variants locked in the dimer and/or tetrameric state.***A*, location of clinical variants displayed on the G6PD tetramer (PDB 6E08). *B*, P-Native PAGE quantification for clinical variants (*n* ≥ 3 for each mutant). The residues for each clinical variant were displayed as spheres and color coded according to the percent activation over the nonlocked version (PDB 6E08). *C*, the dimer is displayed for dL variants and (*D*) the tetramer is displayed for dLtL variants (*n* ≥ 3 for each mutant). The thermostability of G6PD^WT^ compared to (*E*) G6PD^K275N^ (*n* = 3 for each mutant) and (*F*) G6PD^Canton^ (*n* = 3 for each mutant). For P-Native PAGE, differences between mutant and G6PD^WT^ were detected for each group of oligomeric species *via* four one-way ANOVAs (*p* value < 0. 05 for each ANOVA). G6PD, glucose-6-phosphate dehydrogenase.

We next questioned whether biophysical and biochemical properties observed for G6PD^K403Q^ and Class I variants were unique to these mutants and whether clinically relevant mutants could be activated or stabilized *via* the induction of oligomers. To answer this, several clinical variants falling into different clinical classes and located throughout different regions of the protein structure were examined, as shown by the color coding of variants on the G6PD tetramer (Fig. 6*A*). G6PD^Canton^, G6PD^Mediterranean (MED)^, G6PD^Kaiping^, and G6PD^A-^, four of the most common G6PD variants, were included in our study.

Using P-Native PAGE, we found that most Class II, III, IV variants dissociated to a monomer, like G6PD^WT^, in contrast to that observed for G6PD^K403Q^ and Class I variants (Fig. 6*B*), confirming that a dysfunctional dimer is unique to Class I variants and the Class-I like variant, G6PD^K403Q^. Since G6PD^K403Q^ had improved catalytic activity following induction of the tetrameric state, we hypothesized that inducing the tetramer would also improve biochemical parameters for Class I mutants and that this activation would not occur with induction of the dimeric state. Additionally, we hypothesized that induction of the dimeric state and/or tetrameric state may improve catalytic activity and/or stability of other clinical variants. To test our hypotheses, we made double and/or triple mutants for each clinical variant depicted in Figure 6*A* and measured the catalytic activity of each mutant series (Fig. S9, *A*–*M*).

The change in catalytic activity from the nonlocked (nL) enzyme, the normal mutant enzyme, as a function of the position of the mutation is depicted onto the three-dimensional structure for both the dimer-locked and tetramer-locked variants (Fig. 6, *C* and *D*). Interestingly, dimer locking did not improve activity for Class I, II, and III variants tested with one exception; G6PD^A-^, a Class III double variant, had ∼60% activation (Fig. S9, *A*–*M*). In contrast, Class I variants had significant improvements in catalytic activity when locked in the tetrameric state (Fig. S9, *A*–*G*). In fact, activation *via* tetramer locking was specific for Class I variants, as tetramer locking did not improve the catalytic activity of most Class II and III variants; only G6PD^MED^, a Class II variant, exhibited a twofold increase in activity (Fig. S9, *H*–*M*). Consistent with our findings that Class I mutations at the structural NADP^+^-binding site benefit from tetramer stabilization, G6PD^W509A^, a nonclinical variant located on the C-terminal tail, which sandwiches the structural NADP^+^-binding site (19), also exhibits activation by tetramer locking (Fig. S9*D*).

AG1, a small-molecule activator of Class II, III, and WT that stabilizes the dimer state (18) did not significantly improve the catalytic activity of Class I variants (Fig. S9*N*). This is consistent with our data about the lack of benefit of increased dimerization on Class I variants and lack of structural NADP^+^ binding. Interestingly, select variants were inhibited by tetramer locking; G6PD^E274K^ and G6PD^R227W/Q^. It is possible that tetramer locking with a disulfide bond causes steric clashing at the tetramer interface, suggesting that linker length may be an important determinant of activation.

Although dimer locking did not affect catalytic activity for most mutants, it stabilized Class I and Class II variants (Fig. 6, *E* and *F*). The effect on thermostability for various classes of clinical variants varied. For example, G6PD^K275N^, a Class I variant, had a 2.2 °C increase when dimer locking (G6PD^K275NdL^), and an additional 3.1 °C increase when tetramer locking (G6PD^K275NdLtL^), bringing G6PD^K275NdLtL^ T_1/2_ to only 0.7 °C below that of G6PD^WT^ (Fig. 6*E*). Interestingly, G6PD^Canton^ was stabilized by dimer and tetramer locking, despite lack of catalytic activation. G6PD^Canton^, a Class II variant, had a 4.0 °C increase in T_1/2_ when dimer locking (G6PD^CantondL^), with tetramer locking (G6PD^CantondLtL^) adding an additional 3.6 °C to the T_1/2_, bringing the G6PD^CantondLTL^ T_1/2_ to 5.2 °C above that of G6PD^WT^ (Fig. 6*F*). Stabilizing the dimer/tetramer may be a therapeutic strategy to improve stability for catalytically competent mutants, such as Class II or III, with tetramer stabilization being beneficial to Class I variants by improving both catalytic activity and stability.

## Discussion

In our study, we show G6PD^K403Q^ and Class I mutations at and distant from the structural NADP^+^ are impaired in their ability to bind structural NADP^+^, resulting in a dysfunctional dimer with modified biophysical properties; variants with this dimer have low activity and stability, are less prone to dissociation, and are unable to form a tetramer. Although these observations were striking, the structural mechanism remained unclear. Therefore, we solved the protein structure for G6PD^K403Q^ and confirmed that major structural alterations occur in the G6PD^K403Q^ dimer: the structural NADP^+^ is not bound, the catalytic G6P-binding site is occluded, and there are major changes in the globular structure of the dimer. These observations explain our biochemical data; G6P occlusion hinders G6PD activity, and dimer distortion prevents salt-bridge interactions at the tetramer interface, and these findings are consistent with previous structures solved for Class I variants (26).

Although we cannot definitively claim that differences in mobility observed on P-Native PAGE are the direct result of structural changes observed *via* crystallography, our structural data combined with the fact that a dysfunctional dimer is unique to Class I and Class I-like variants (Class II, III, and IV variants did not form a dysfunctional dimer) suggest that P-Native PAGE can distinguish between G6PD, which can or cannot bind structural NADP^+^. Furthermore, our findings using G6PD^K403Q^ are physiologically relevant, since the G6PD^K403Q^ mutation mimics *in vivo* acetylation of G6PD^WT^ (12); acetylation at K403 likely induces the formation of a dysfunctional dimer, which may be used as a regulatory mechanism to inactivate/activate G6PD^WT^ during cellular oxidative stress.

To better understand functional differences between dimeric and tetrameric G6PD, we used synthetic mutants locked in the dimer and/or tetrameric state and found that the tetramer is more active and stable than the dimer. We used the same strategy, dL and dLtL in combination with pathogenic mutations, and found that stabilizing the dimer can recover protein stability in pathogenic variants, regardless of clinical classification. Importantly, we found that inducing tetramerization offers improvement in the activity of Class I and Class I-like variant activity, but not Class II or III, and further improves stability for all variants, regardless of clinical classification. Two important exceptions should be noted: dimer stabilization improved activity for the G6PD^A-^ variant, and tetramer stabilization improved activity for the G6PD^Med^ variant. These observations have important clinical implications as the G6PD^A-^ mutation is the most common African mutation (27), affecting 5 to 7% of all African males (2), and G6PD^Med^ is the most prevalent variant in the Middle East (28) and highly prevalent in India (29). These data suggest that compounds such as AG1 may be particularly useful to protect patients with these mutations.

**Figure 7:**
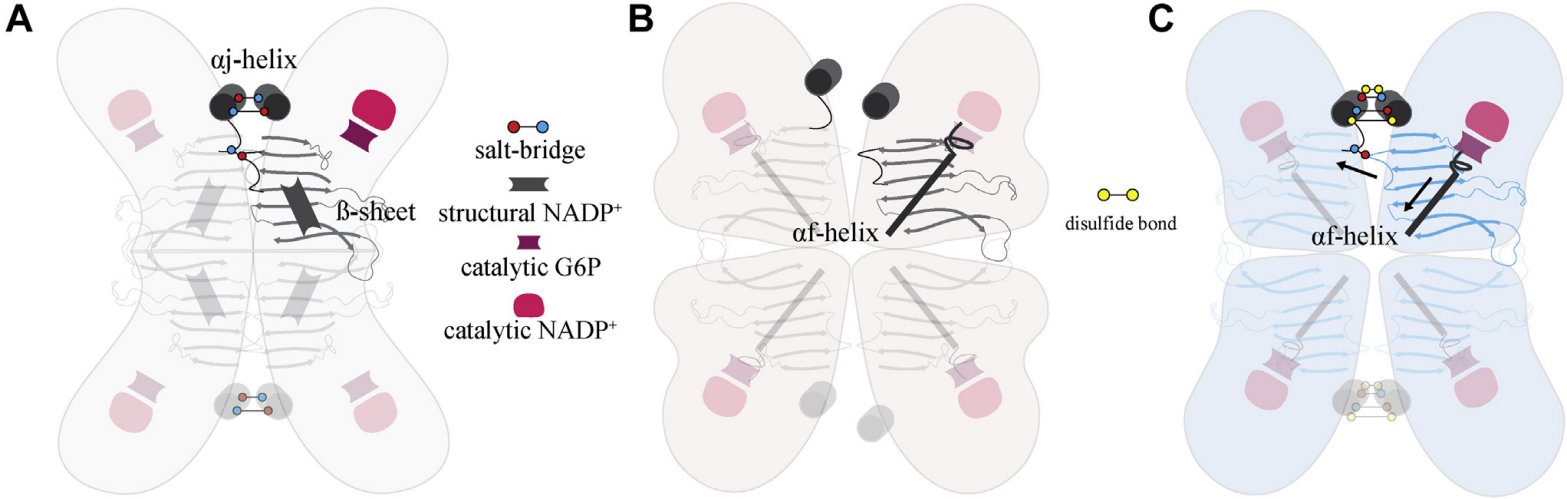
**Model for recovery of activity and stability for Class I variants *via* tetramer stabilization.** Important structural features in the (*A*) G6PD^WT^ and (*B*) G6PD^K403Q^ mutants and (*C*) model for recovery of G6PD^K403Q^ activity. G6PD^A277C^ forms a disulfide bond across αj-helix, likely stabilizing αj-helices. This likely prevents K275/E347 disruption and ß-sheet from shifting. Stabilization of the ß-sheet may prevent the αf-helix from occluding the G6P pocket.

Combining our findings with the structural insight from Horikoshi *et al.*, we propose a model for how inducing the tetramer improves activity of Class I mutations (Fig. 7). As A277C is located at opposing ends of the αj-helix, tetramer locking through disulfide bond formation likely prevents the αj-helix from shifting, which maintains the K290/E287 and E347/K275 salt bridges, reducing shifting of the structural NADP^+^ ß-sheet. By restoring αj-helix positioning and the corresponding E347/K275 interaction, the enzymatic activity of Class I may be improved due to the removal of the occlusion of the G6P binding pocket by the αf loop. Loss of interactions at the tetramer interface appears to be most detrimental when structural NADP^+^ is not bound. Mutation of the αj-helix loop salt bridge to the structural NADP^+^ has different effects, depending on which amino acid residue was mutated; G6PD^E347A^ had 60% of G6PD^WT^ activity and did not form a dysfunctional dimer (Fig. 4, *H* and *I*), whereas G6PD^K275N^ had <10% of G6PD^WT^ activity and formed a dysfunctional dimer (Fig. 2, *G* and *H*). It is likely that when structural NADP^+^ is bound, NADP^+^ binding is sufficient to stabilize the ß-sheet, making loss of tetramerization less severe. However, if structural NADP^+^ is not bound, loss of this salt-bridge interaction may allow for ß-sheet shifting.

We suggest that the key contribution of “locking” the enzyme in the tetrameric state is increasing protein stability for variants of different classifications. Increased protein stability is particularly relevant to the clinical impact of G6PD^def^, as RBCs lack nuclei, and therefore, proteins must maintain proper stability for the entire life span of the RBCs (60–90 days). Consequently, as RBCs age, their residual G6PD activity is largely dependent on protein levels. We therefore suggest that improving the stability of any mutant with lower than WT catalytic activity, or in the case of Class I, a significant improvement in stability combined with a small increase in activity, may be sufficient to improve the clinical phenotype; clinical observations suggest that a shift from Class I to Class II-like state will result in a less severe phenotype (4).

Although the identification and development of a tetramer stabilizer for G6PD may be challenging, molecular chaperones that stabilize dimeric actin (30), tetrameric transthyretin (31, 32, 33, 34, 35), and even pentameric S100A4 protein complexes (36) have been discovered, with some used in the clinic. In the case of transthyretin, a symmetric small molecule stabilizes the tetramer interface of an unstable V122I clinical variant (33, 37). For S100A4, two trifluoperazine molecules help form a pentamer of dimers of S100A4 using a protein surface distinct from the dimer interface or its crystal packing interfaces (36). Both examples could be relevant in designing small molecules or peptides that will induce and stabilize the tetrameric state of G6PD mutants. Future work will focus on the identification of such small molecules or peptides that correct the dimer distortion and induce G6PD tetramerization.

## Experimental procedures

### Materials

Chemicals: NADP^+^ (Amresco – 0760), NADPH (Millipore Sigma – 10107824001), Glucose-6-phosphate (CHEM-IMPEX International – INC 00866), resazurin (ACROS Organics – AC189900050). Reagents: TALON Superflow resin (cytiva – 28957502), High Purity Bovine Thrombin (BioPharm Laboratories – 91-030-006), protease inhibitor cocktail (Millipore Sigma – p8340), Diaphorase (Worthington Biochemical Corporation – LS004330). Antibodies purchased from Everest Biotechnology, G6PD (aa 308–320) goat (Everest Biotech – EB07841). Analytical SEC column: Superdex 75 10/300 Gl (GE Healthcare – 17-5174-01). Purification SEC column: Superdex 200 HiLoad 16/600 (GE Healthcare Life Sciences). SEC-SAXS column: Superdex 200 Increase PC 3.2/300 column (Cytiva).

### Cloning

G6PD was inserted into a pET-28a (+) vector, between *Nde* I and *Sal* I restriction sites, with a stop codon added at the C-terminal end before the C-terminal his-tag. For mutants, site-directed mutagenesis was performed using the Agilent QuikChange II kit.

### Protein expression and purification

G6PD-pET-28a (+) was transformed into OverExpress C43 (DE3) chemically competent *E. coli* cells. Cells were grown in TB medium, and protein expression was induced with IPTG at 28 °C for 24 h. Cell lysate was incubated with TALON Superflow resin for 1 h at 4 °C. Following resin washing, protein was eluted *via* overnight Thrombin cleavage at 4 °C to remove the N-terminal his-tag. For X-ray crystallography and SEC-SAXS experiments, protein was subjected to further purification on a Superdex 200 HiLoad 16/600 (GE Healthcare Life Sciences) column in 50 mM Tris (pH7.4), 150 mM NaCl, 10 mM G6P.

### Activity assays

(1) Activity assay for biochemical characterization of clinical mutants relative to G6PD^WT^: 3 nM recombinant G6PD was incubated in buffer containing 50 mM Tris (pH 7.4), 10 mM MgCl_2_, 200 μM NADP^+^, 600 μM G6P, and 0.005% Tween20. G6P was added to start the reaction and NADPH absorbance at 340 nm was measured for 5 min at 23 °C. The slope (ΔAbs/ΔTime) was used to compare enzyme activity for mutants, with data normalized to G6PD^WT^ activity. (2) Michaelis–Menten kinetics for G6PD^WT^, G6PD^E347A^, G6PD^A277C^: Activity assay 1 conditions were used, however, to prevent NADP^+^ depletion, 1 U/ml diaphorase and 0.1 mM resazurin fluorescence were used (NADPH conversion to NADP^+^ is coupled to resorufin production). The excitation/emission at 565 nm/590 nm respectively was measure and converted to NADPH/sec with an NADPH standard curve. Data were fit to the Michaelis–Menten equation in GraphPad Prism. (3) Activity assay for G6PD^K403Q^ locked in dimer and/or tetrameric state: Activity assay 1 conditions were used; however, enzyme concentration was increased to 70 nM so relative difference in activity could be accurately assessed. For the condition with low NADP^+^, NADP^+^ was reduced to 10 μM. (4) Michaelis–Menten Kinetics for G6PD^K403Q^ series: Activity assay 2 conditions were used; however, enzyme concentration was increased to 70 nM so differences in activity could be accurately assessed. (5) Activity assay pathogenic variants locked in dimer and/or tetrameric state: Activity assay 3 conditions were used at 10 μM NADP^+^; however, 70 nM of Class I variants protein was used, while 6 nM of less severe variant protein was used. Activity assay for AG1: 3 nM recombinant enzyme was incubated in buffer containing, 50 mM Tris (7.4), 3.3 mM MgCl2, 10 μM NADP^+^, 100 μM G6P, 0.010% Tween20, 0.5 mM EDTA, 1 U/ml diaphorase, and 0.1 mM resazurin. In total, 100 μM AG1 was incubated with mixture for 5 min at 23 °C. G6P was added to start reaction, and fluorescence was measured and slopes used to compare activity between series. These conditions were established by Hwang *et al.* (18) for AG1.

### Thermostability assay

In total, 333 nM recombinant enzyme was incubated in 50 mM Tris (pH7.4), 150 mM NaCl, and 5 μM NADP^+^ for 20 min at various temperatures. Enzyme activity, after temperature incubation, was measured according to conditions described in activity assay 1. The reaction rate was normalized to the maximum activity for a given temperature interval, and the normalized activity was plotted and fit to the Boltzmann sigmoidal equation in GraphPad Prism. The temperature at which the enzyme maintained half activity (T_1/2_), or the melting temperature, was generated for each mutant. Unique temperature intervals were used for independent experiments, in triplicates, and temperature intervals were combined to generate three Boltzmann sigmoidal fits, with each fit representing one triplicate.

### Analytical SEC

All analytical SEC experiments were performed on a Superdex 75 10/300 Gl column and ran at a flow rate of 0.4 ml/min. (1) SEC for G6PD^K403Q^ and Class I variants *versus* G6PD^WT^: 500 nM recombinant G6PD was incubated in 80 mM NaP (pH 8.0) buffer with and without 10 μM NADP^+^ for 30 min at 23 °C. The protein was run on FPLC using 80 mM NaP (pH 8.0). (2) Monomer condition: 1 μM recombinant G6PD was incubated in 50 mM Tris (pH7.4), 150 mM NaCl, and 10 mM G6P for 30 min and ran on FPLC using 50 mM Tris (pH 7.4), 150 mM NaCl. (3) Tetramer condition: 1 μM recombinant G6PD was added to buffer containing 80 mM NaP pH 6.0, 20 mM MgCl2, 10 μM NADP^+^, 20 μM EDTA, 0.005% Tween20. For SEC chromatograms, data were normalized to total peak area, followed by normalization to the data maxima, so that chromatograms have a maximum absorbance of ∼1.

### P-native PAGE

Laemmli sample buffer devoid of SDS and BME was added to samples. The sample was not boiled and instead immediately ran on SDS-PAGE. (1) P-Native PAGE: 850 nM protein was incubated in 50 mM Tris (pH 7.4), 150 mM NaCl, at 23 °C for 30 min followed by P-Native PAGE and western blotting. P-Native blots were quantified using Fiji (2) Cross-linking followed by P-Native PAGE: For Figure 4*E*, 270 nM G6PD was incubated in tetramer conditions (see Analytical SEC Methods for conditions) for 45 min at 23 °C. Reaction was cross-linked with 0.4% glutaraldehyde for 20 min and quenched with 1M Tris (pH 8.0). Sample was run using P-Native PAGE followed by Western blotting. The same assay was used in Figure 4*B*; however, the incubation buffer was 50 mM Tris (pH 7.4), 150 mM NaCl.

### Crystallization of G6PD^K403Q^ and G6PD^K403QdLtL^ dimer

For G6PD^K403Q^, 1 μl of 1 mM AG1, 2 mM NADP^+^, 8 mg/ml G6PD^K403Q^ were mixed and incubated with 1 μl 100 mM MES pH 6.5, 13% PEG8000, 250 mM MgCl_2_, 1.4% 1,6-hexanediol and incubated at 20 °C using the sitting drop vapor diffusion method. For G6PD^K403QdLtL^ dimer, 2 μl of 1 mM AG1, 2 mM NADP^+^, 5 mg/ml G6PD^K403QdLtL^ dimer were mixed and incubated with 2 μl 100 mM MES pH 6.5, 12% PEG4000, 60 mM MgCl_2_, and incubated at 20 °C using the hanging drop vapor diffusion method.

### Data collection, structure solution, and refinement

The diffraction data of G6PD^K403Q^ and G6PD^K403QdLtL^ were collected at 100˚K at the BL12-2 of the Stanford Synchrotron Light Source using Pilatus 6M detectors. Crystals for the G6PD^K403Q^ were cryo cooled using well solution supplemented with 20% glycerol. Crystals of the G6PD^K403QdLtL^ dimer were cryo cooled using well solution supplemented with 30% ethylene glycol. We collected 360 degrees of data with 0.2 degree per image. Diffraction data were processed with XDS suite of programs (38). The structure of G6PD^K403Q^ was solved by molecular replacement with Phaser (39) by using coordinates of the F381L mutant (PDB 6VA8) as the search model. The structure of G6PD^K403QdLtL^ dimer was solved by molecular replacement with AMoRE (40) by using coordinates of P396L mutant (PDB 6VA7) as the search model. Iterative rounds of model building and refinements were performed using Coot (41) and refmac (42) programs. The details of data collection and refinement are given in Table S1.

### SEC-SAXS

SEC-SAXS experiments were performed at SSRL beamline 4-2 as the previous report with some modifications (26). G6PD^K403QdLtL^ was purified according to the protein purification section above, and 10 mg/ml of purified G6PD^K403QdLtL^ was incubated with 10 mM G6P for ≥ 30 min. SEC-SAXS data were collected using Superdex 200 Increase PC 3.2/300 column (Cytiva) using 50 mM Tris pH 7.4, 150 mM NaCl, 10 mM G6P running buffer. In total, 500 images were collected with 1-s exposure every 5 s at a 0.05 ml/min flow rate. After the 100th image (blank data collections), the X-ray shutter was closed until just before the sample was eluted to keep the sample cell clean from debris formed by X-ray radiation. An additional sample cell wash was executed during the shutter closure. Data reduction and initial analyses were performed using the BL4-2 automated SEC-SAXS data processing and analysis pipelines, *SECPipe* (https://www-ssrl.slac.stanford.edu/smb-saxs/node/1860). It implements the program *SASTOOL* (https://www-ssrl.slac.stanford.edu/smb-saxs/node/1914) and *ATSAS AUTORG* (43). The data were presented as I(q) *versus* q, where q = 4πsin(θ)/λ, 2θ is the scattering angle, and λ is the wavelength of the X-ray. After careful manual inspection, a total of ten images were selected to generate the averaged profile for further analysis. The program *GNOM* was used for the indirect Fourier transform to estimate the distance distribution function P(r) (44). The SAXS modeling program CORAL was employed for reconstructing disordered regions of the crystal structures (25). For the G6PD^K403QdLtL^ tetramer, a subunit of the crystal structure of G6PD^WT^ (PDB 6E08) was used an input model, and disordered N-terminal portion of 31 residues was modeled with P22 symmetry. For G6PD^K403QdLtL^ dimer, a subunit of the crystal structure (PDB 7SEH) was split into three rigid bodies and three disordered regions in the subunit (31, 26, 14 residues at the N-terminus, 407th to 432nd, and C-terminus, respectively) were reconstructed during the modeling with P2 symmetry. Twenty independent runs were performed, and the model with the lowest χ^2^ value was selected as the best model. All models showed that the disordered portions were located virtually at the same position (Figs. S6 and S7, *C* and *D*). The SAXS parameters of the best CORAL models were computed using the program CRYSOL (45). MW was estimated from the Porod volume (MW(Da) = Pv ∗ 0.625) (25). Experimental and analytical details are summarized in Table S2.

### Statistical analysis

In Figure 1, *I* and *J*, G6PD^K403Q^ was compared with G6PD^WT^, and statistical differences were calculated by a two-tailed unpaired *t* test. In, Figure 1*G*, G6PD^K403Q^ was compared with G6PD^WT^, and statistical differences were calculated for each group of oligomeric species (dependent variables) independently, using four two-tailed unpaired t tests. In Figures 2, *C* and *G*, 4*H*, and 6*B*, mutants were compared with G6PD^WT^, and statistical differences were calculated for each group of oligomeric species (dependent variables) independently by four one-way ANOVAs, Dunnett’s multiple comparisons test. In Figure 2, *D* and *H*, mutants were compared with G6PD^WT^, and statistical differences were calculated by ordinary one-way ANOVA, Dunnett’s multiple comparisons test. In Figures 4, *I* and *K*, 5, *A*, *B* and *D* and 6, *E* and *F*, and Fig. S9, *A*–*M*, statistical differences between mutants were calculated by ordinary one-way ANOVA, Tukey’s multiple comparisons test. For Fig. S9*N*, statistical differences between the no treatment group and AG1-treated group were calculated *via* a two-way ANOVA, Šidák’s multiple comparisons test. For all plots with bars displayed and K_m_ tables, error bars represent mean ± standard deviation. Asterisks symbolize *p* values: n.s *p* > 0.05, ∗ *p* ≤ 0.05, ∗∗ *p* ≤ 0.01, ∗∗∗ *p* ≤ 0.001, ∗∗∗∗ *p* ≤ 0.0001, and *n* represents a biological replicate, where each *n* was performed independently. For thermostability assays, measurements were derived from multiple independent experiments, performed in triplicates. Independent measurements were combined to generate a Boltzmann sigmoidal fit, with *n* representing triplicates and error representing standard deviation among the triplicate fit values.

## Data availability

Protein structures have been deposited in the Protein Data Bank (PDB) with accession codes 7SEI, 7SEH for G6PD^K403Q^, G6PD^K403QdLtL^ dimer, respectively. All other data are included in the manuscript.

## Supporting information

This article contains supporting information.

## Conflict of interest

The authors declare that they have no conflicts of interest with the contents of this article.
