# Supplementary material for: Stabilization of glucose-6-phosphate dehydrogenase oligomers enhances catalytic activity and stability of clinical variants
Source: J Biol Chem. 2022 Jan 20;298(3):101610. doi: 10.1016/j.jbc.2022.101610 (PMC8861134; doi:10.1016/j.jbc.2022.101610)
Supplement: Supplemental Figures S1–S11; Tables S1 and S2 [file mmc1.pdf]

Stabilization of glucose-6-phosphate dehydrogenase oligomers enhances catalytic activity and stability of clinical variants

Adriana Ann Garcia[1], Irimpan I. Mathews[2], Naoki Horikoshi[3,4,5], Tsutomu Matsui[2], Manat Kaur[5], Soichi Wakatsuki[4,5*], Daria Mochly-Rosen[1*]

[1]Department of Chemical and Systems Biology, School of Medicine, Stanford University, Stanford, CA, USA; [2]Stanford Synchrotron Radiation Lightsource, SLAC National Accelerator Laboratory, Menlo Park, CA, USA; [3]Life Science Center for Survival Dynamics, University of Tsukuba, Tsukuba, Ibaraki, Japan; [4]Biological Sciences Division, SLAC National Accelerator Laboratory, Menlo Park, CA, USA; [5]Department of Structural Biology, School of Medicine, Stanford University, Stanford, CA, USA

*For correspondence: soichi.wakatsuki@stanford.edu (Soichi Wakatsuki), mochly@stanford.edu (Daria Mochly-Rosen)

Running Title: Stabilizing G6PD oligomers improves G6PD function

This document includes supplementary Figures S1-S11; Tables S1 and S2; Movie S1-S6 figure legends; Experimental procedures

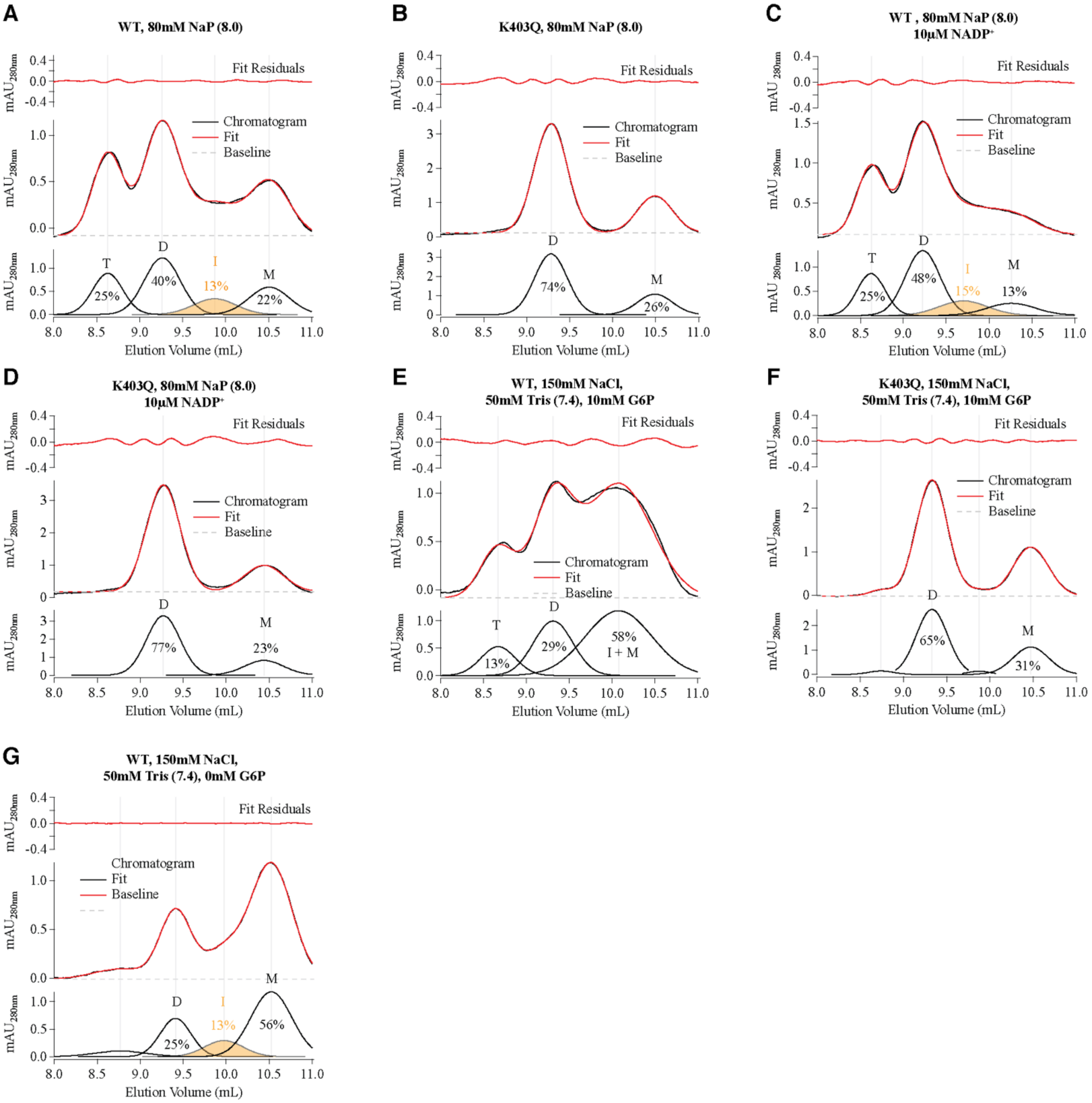

Figure S1. SEC peak deconvolution and quantification of G6PD^{WT} and G6PD^{K403Q} in different buffer conditions. *A-B*, 80mM NaP (pH 8.0). *C-D*, 80mM NaP (pH 8.0), 10µM NADP^{+}. (E-F) 50mM Tris (pH 7.4), 150mM NaCl, 10mM G6P and (*G*) without G6P. Each graph is one dataset quantified.

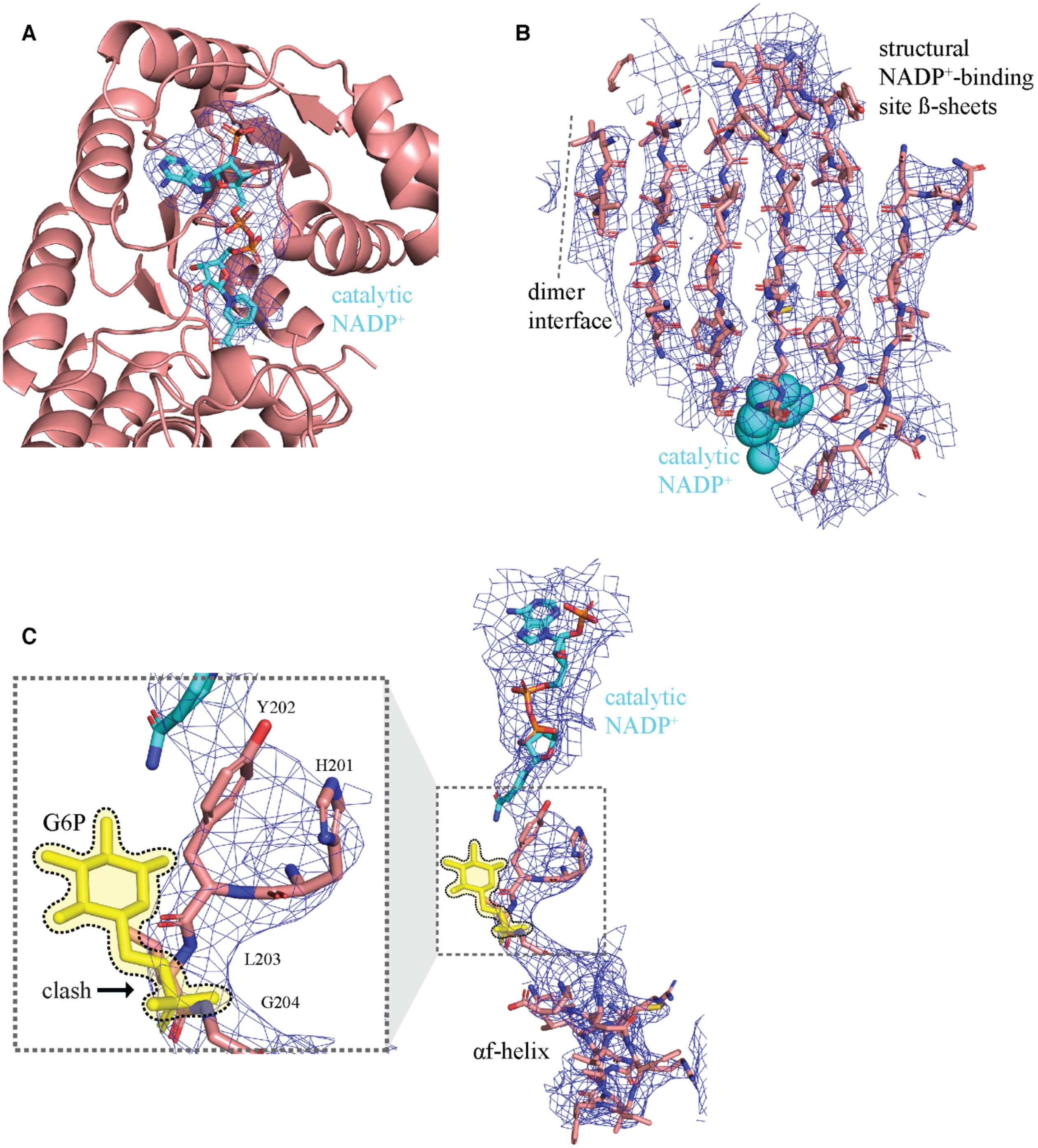

Figure S2. Electron density maps for G6PD^{K403Q} (PDB 7SEI). *A*, the OMIT map for the catalytic NADP^{+} contoured at 2.5 sigma and superimposed with the model of NADP^{+}. The 2Fo-Fc map for the (*B*) structural NADP^{+}-binding site (*C*) and the αf-helix contoured at 1.0 sigma and superimposed with the structural model for the corresponding regions. For (*B*) part of the catalytic NADP^{+} is displayed as spheres to orient the viewer. For (*C*), the G6PD^{K403Q} dimer was aligned to a G6PD^{WT} monomer (PDB 2BHL) and G6P from 2BHL was displayed in yellow against G6PD^{K403Q} 7SEI in salmon.

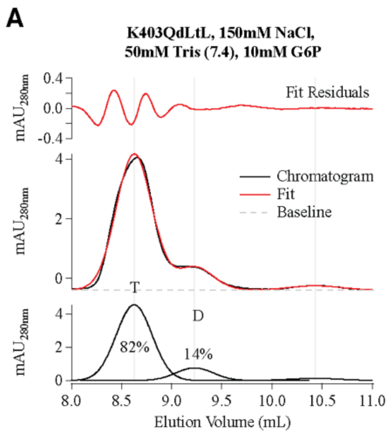

Figure S3. *A*, SEC peak deconvolution and quantification of G6PD^{K403QdLtL} with 10 mM G6P. One representative dataset quantified.

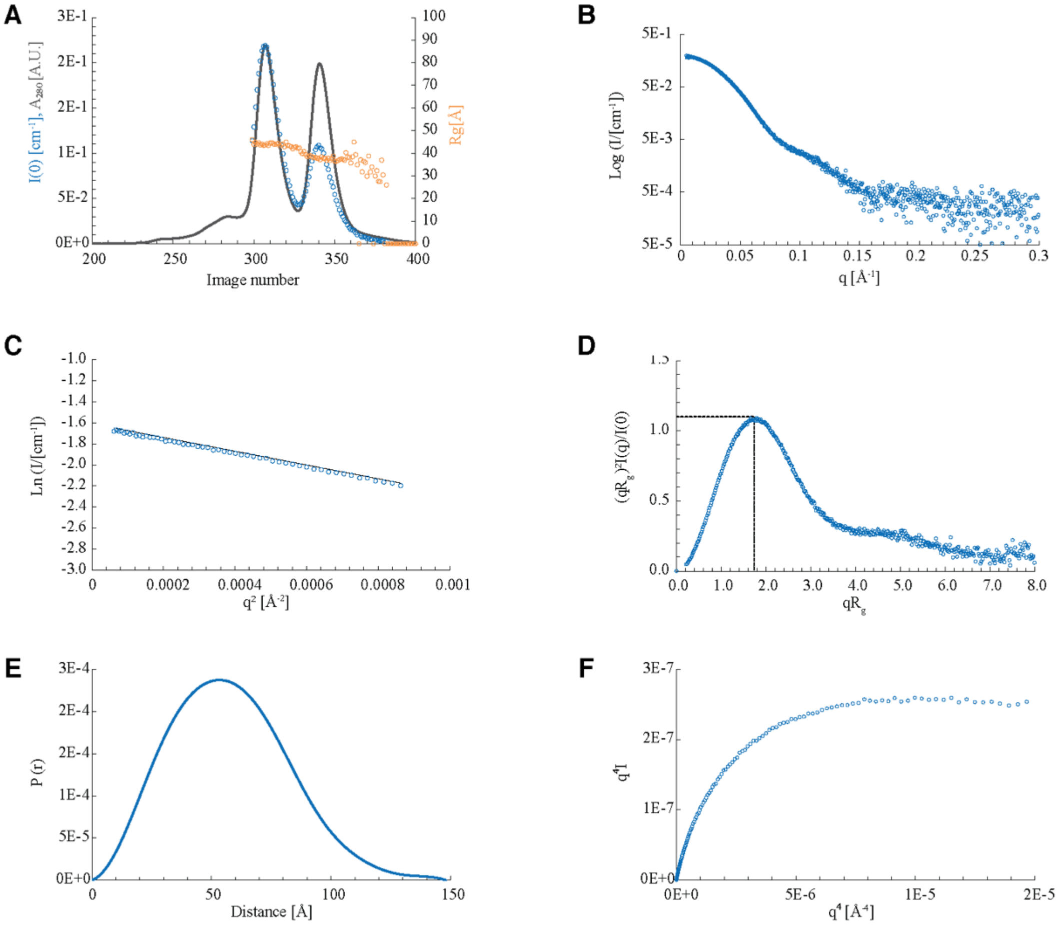

Figure S4. SEC-SAXS plots for G6PD$^{\mathrm{K403QdLtL}}$ tetramer fraction. *A*, the SEC-SAXS profile. *B*, I(q) vs. q as log-linear plots. *C*, *Guinier* plots. Open circles and black lines indicate experimental data and *Guinier* fit, respectively. *D*, Dimensionless Kratky plots. *E*, P(r) functions. *F*, *Porod-Debye* plots.

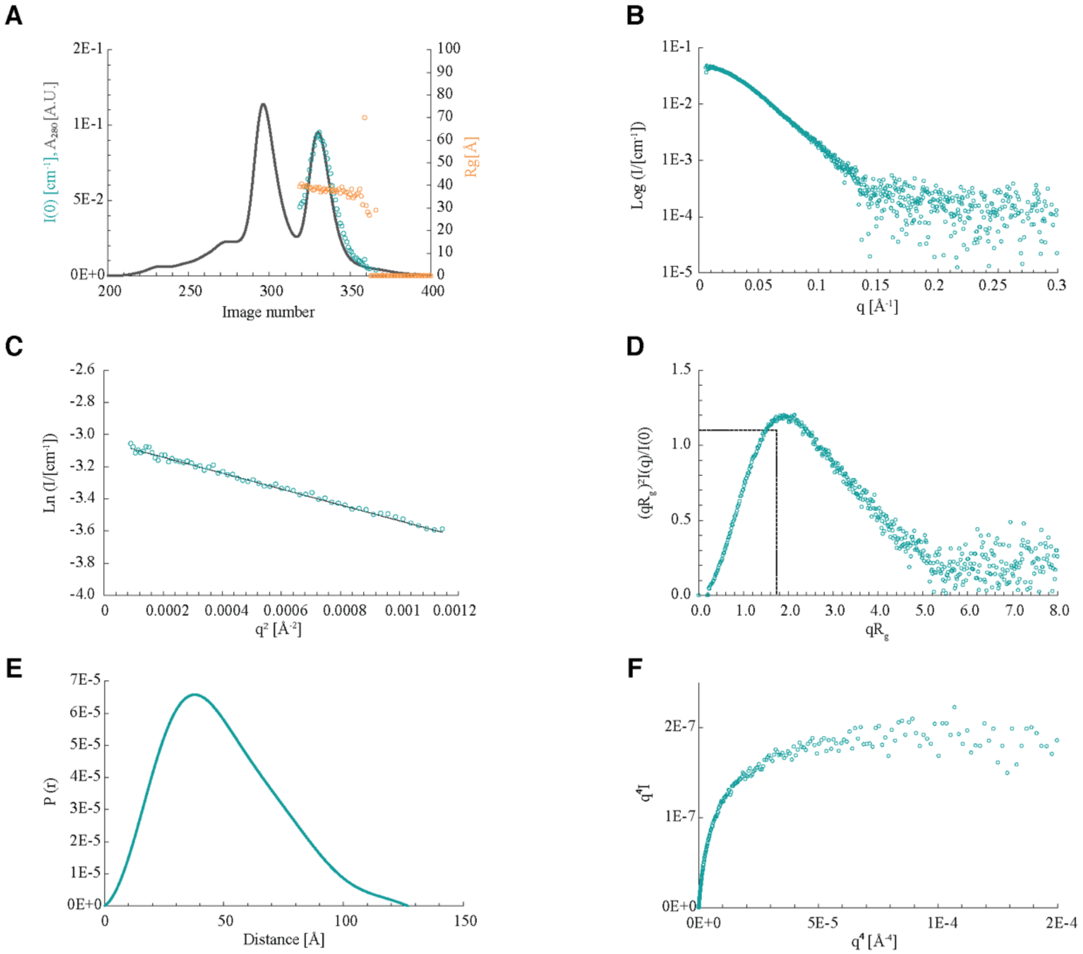

Figure S5. SEC-SAXS plots for G6PD^{K403QdLtL} dimer fraction. *A*, the SEC-SAXS profile. *B*, I(q) vs. q as log-linear plots. *C*, *Guinier* plots. Open circles and black lines indicate experimental data and *Guinier* fit, respectively. *D*, Dimensionless Kratky plots. *E*, P(r) functions. *F*, *Porod-Debye* plots.

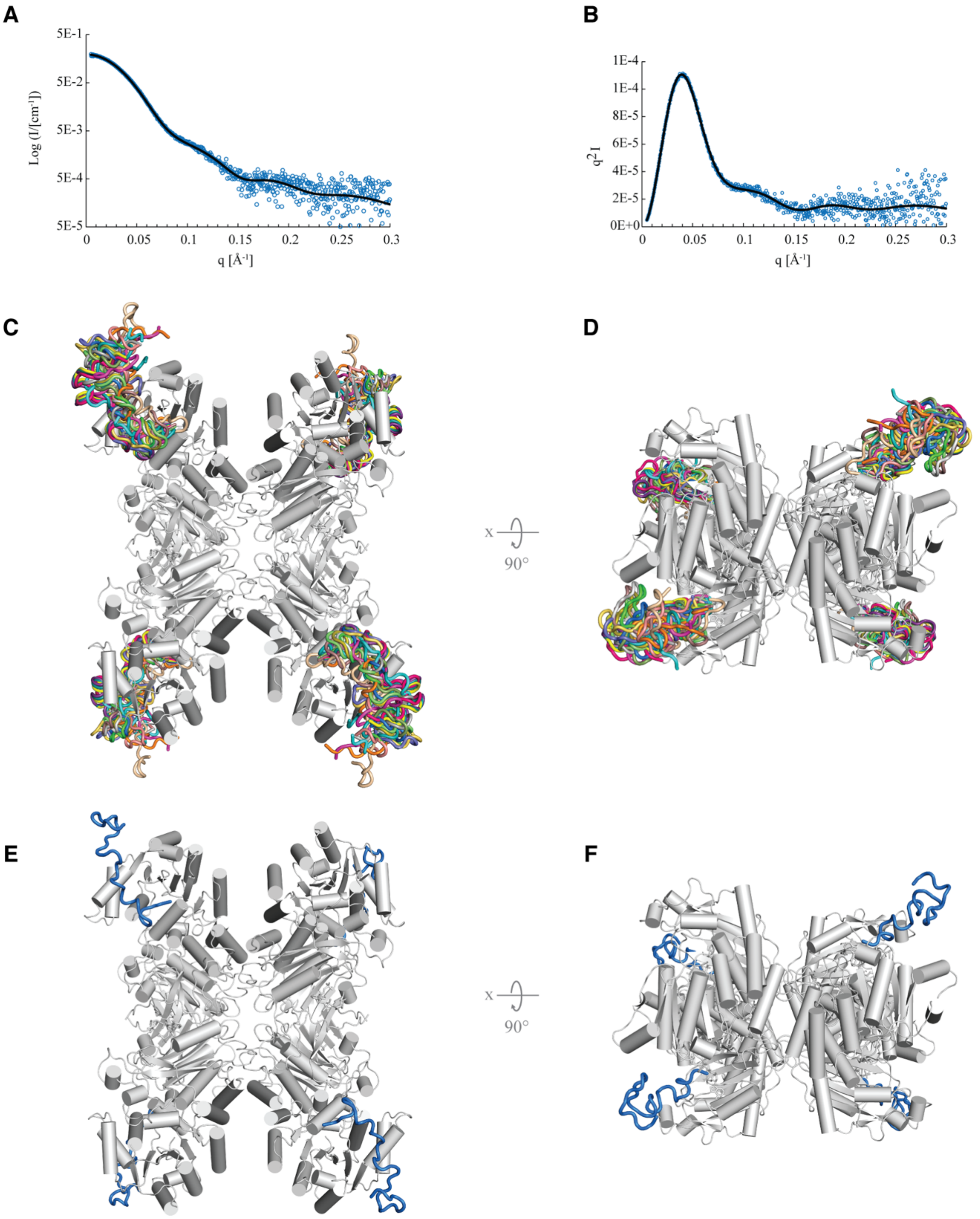

Figure S6. CORAL modeling of the G6PD^{WT} tetramer fit to the experimental SEC-SAXS data for the G6PD^{K403QdLtL} tetramer fraction. *A* and *B*, the theoretical I(q) vs. q and Kratky plot of the G6PD^{WT} (PDB 6E08) best-fit CORAL model (black line) was fitted to the experimental data of G6PD^{K403QdLtL} tetramer (dots). *C* and *D*, 20 runs of CORAL modeling for the G6PD^{WT} (PDB 6E08) tetramer were performed and all 20 structures were superimposed. *E* and *F*, the best-fit model of the G6PD^{WT} tetramer.

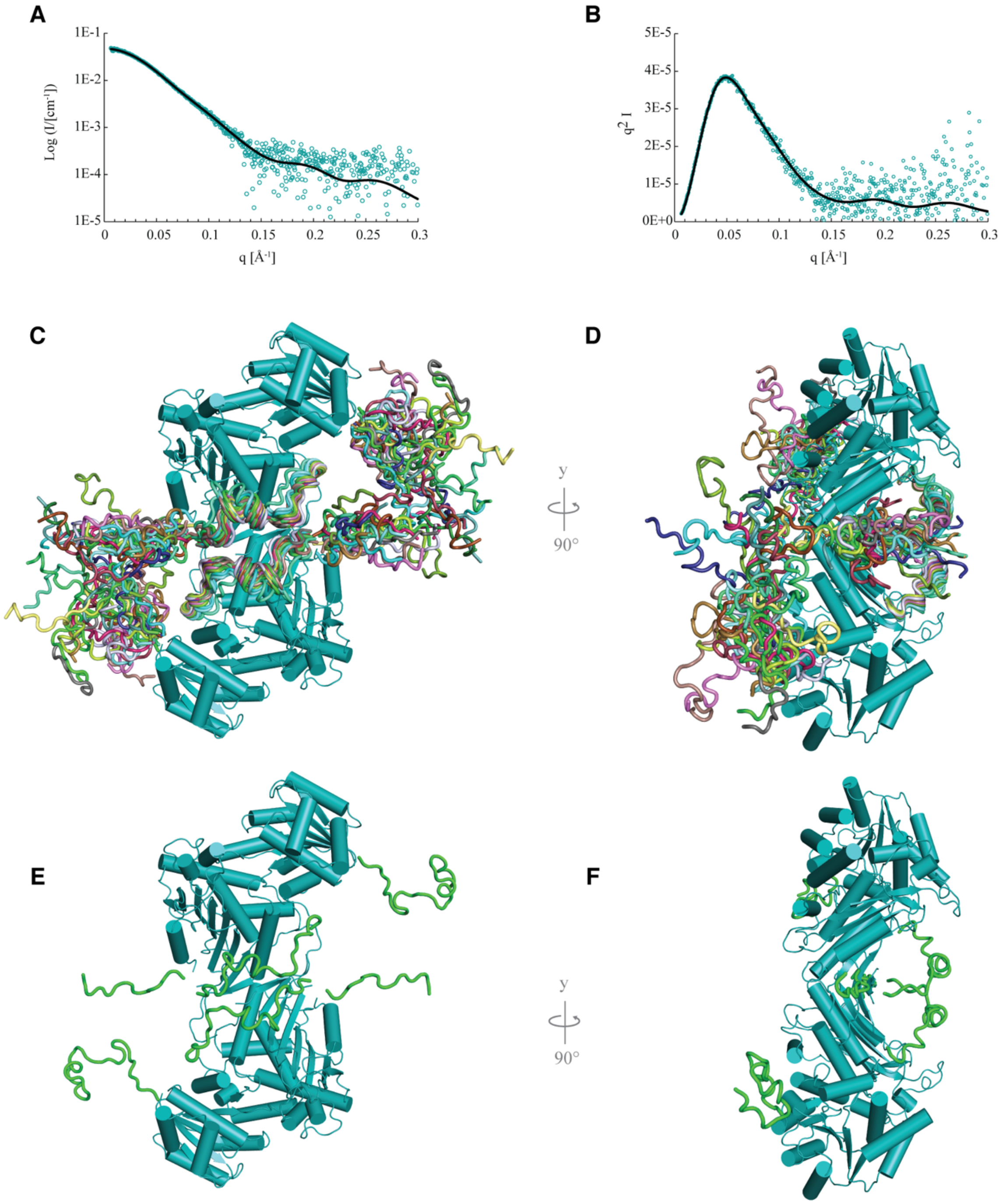

Figure S7. CORAL modeling of the G6PD^{K403QdLtL} dimer fit to the experimental SEC-SAXS data for the G6PD^{K403QdLtL} dimer fraction. *A* and *B*, the theoretical I(q) vs. q and Kratky plot of the G6PD^{K403QdLtL} dimer (PDB 7SEH) best-fit CORAL model (black-line) was fitted to the experimental data of G6PD^{K403QdLtL} dimer (dots). *C* and *D*, 20 runs of CORAL modeling for the G6PD^{K403QdLtL} dimer (PDB 7SEH) tetramer were performed and all 20 structures were superimposed. *E* and *F*, the best-fit model of the G6PD^{K403QdLtL} dimer.

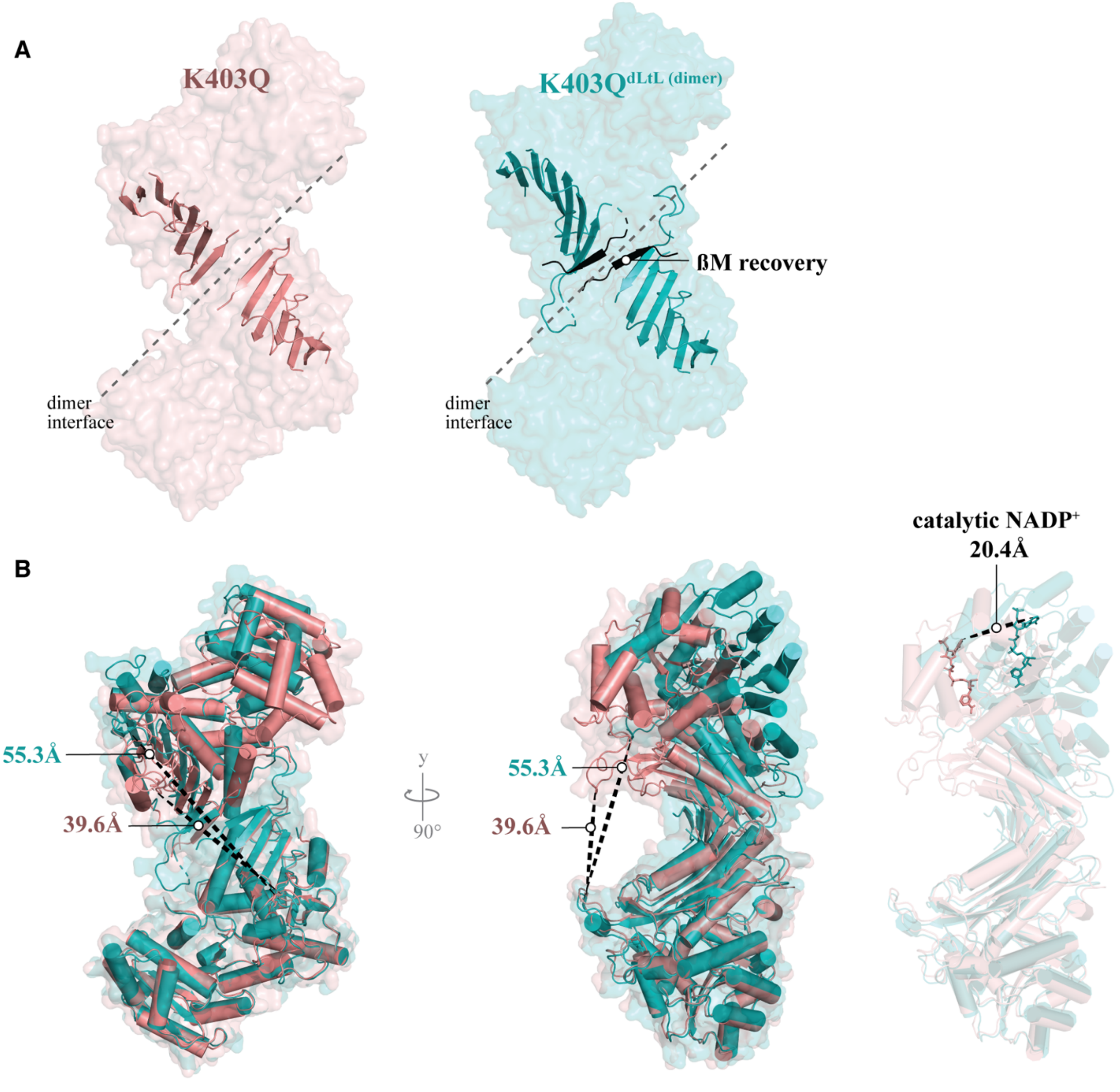

Figure S8. Comparison of G6PD^{K403Q} (PDB 7SEI) and G6PD^{K403QdLtL} dimer (PDB 7SEH) crystal structures. *A*, recovery of the structural NADP$^+$ ßM strand in the G6PD^{K403QdLtL} dimer. B, measurements between G341 G6PD^{K403Q} and G6PD^{K403QdLtL} dimer monomers and the catalytic NADP$^+$.

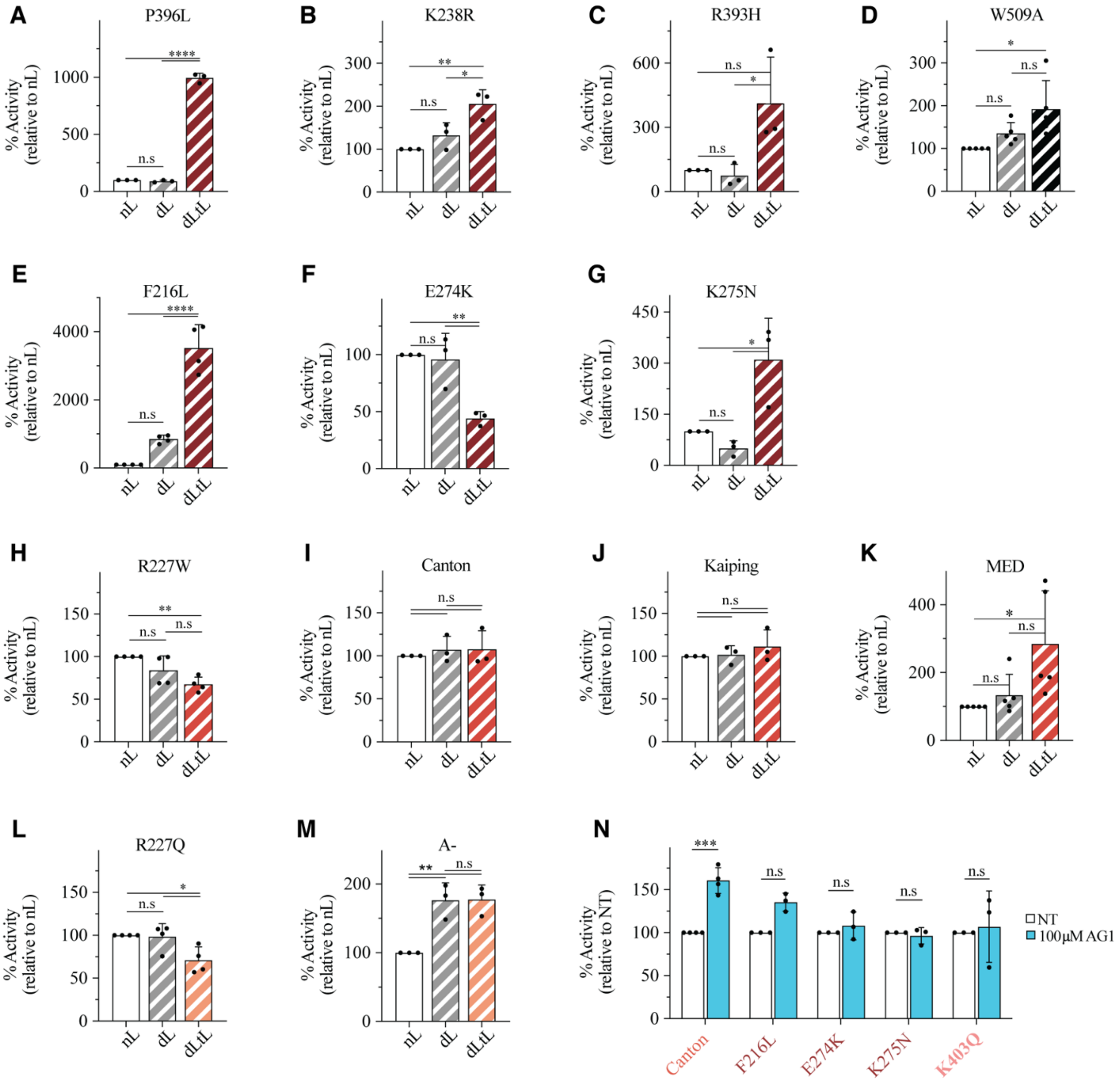

Figure S9. *A-M*, activity of pathogenic variants locked in the dimer and/or tetramer state relative to the non-locked activity ($n \geq 3$ for each mutant). *N*, Class I variant activity with and without AG1, compared to Canton, a Class II variant known to have AG1 activation ($n \geq 3$ for each mutant).

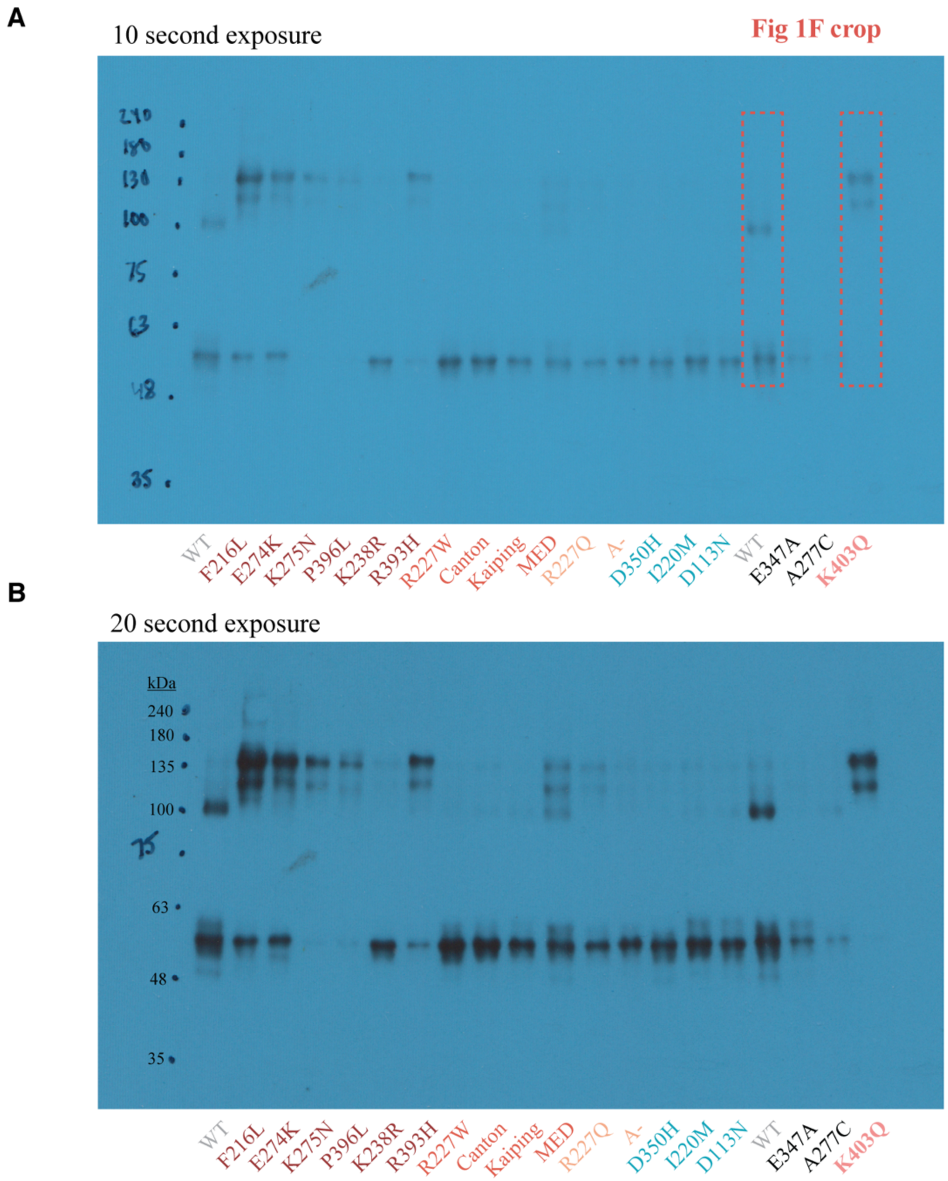

Figure S10. Full western blot for P-Native PAGE assay. One representative western blot is shown at (*A*) 10 and (*B*) 20 second exposures. Dashed boxes indicate the region where data was cropped and cropped data is displayed in Fig. 1*F*.

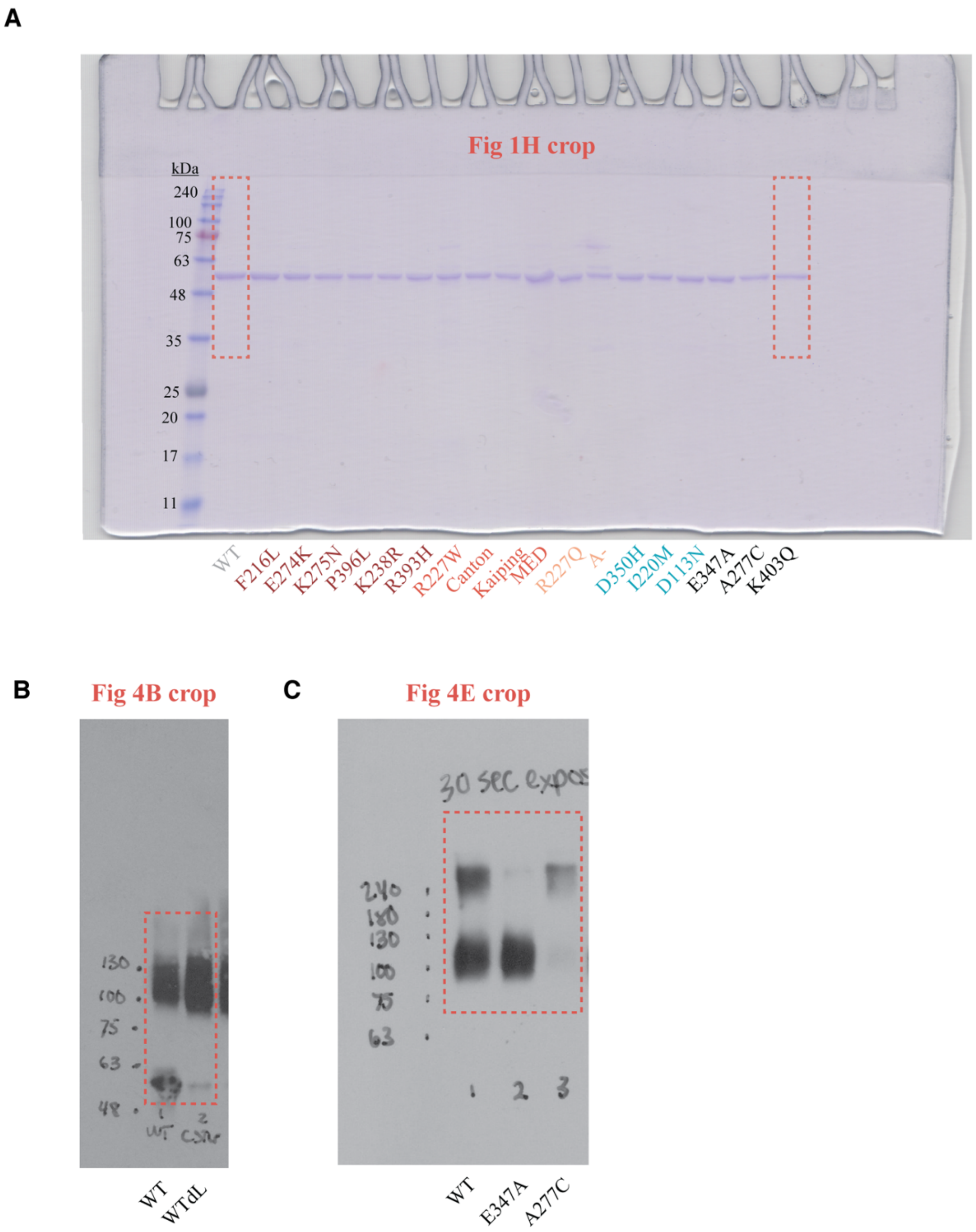

Figure S11. Full Coomassie and western blot for SDS-PAGE and crosslinking western blot assay. Dashed boxes indicate the region where data was cropped and cropped data is displayed in (*A*) Fig. 1*H*, (*B*) Fig. 4*B*, and (*C*) Fig. 4*E*.

	$G6PD^{K403Q}$	$G6PD^{K403QdLtL}$ dimer
**Crystallographic parameters**		
Space group	$P4_12_12$	$P2_12_12_1$
Unit-cell dimensions	157.73Å, 157.73Å, 113.82Å<br>90.0˚, 90.0˚,90.0˚	74.64Å, 87.86Å, 160.68Å<br>90.0˚, 90.0˚,90.0˚
**Data collection statistics**		
Resolution limits (Å)	39.4 – 3.65	38.99-2.90
No: of observed reflections	438256	314920
No: of unique reflections	16483	24109
Completeness overall/outer shell	99.9/100.0	99.9/100.0
CC1/2 (overall/outer shell)	99.9/63.2	99.9/67.1
R_{sym}[a] (%) overall/outer shell & os I/σ	21.7/279.6 & 1.7	20.8/297.8 & 1.5
**Refinement statistics**		
Resolution limits (Å)	39.4-3.65	38.99-2.90
Number of reflections/% (|F|>2σ|F|)	15658/99.9	22903/99.9
Reflections used for R_{free}	825	1206
R_{factor}[b] (%)	19.1	20.6
R_{free} (%)	23.5	30.2
Model contents/average B($Å^2$)		
Protein atoms	3439/147.5	7158/82.7
Ligand	48/136.1	96/104.4
Water molecules	0	37/61.5
RMS deviations		
Bond length (Å)	0.007	0.005
Bond angle (°)	1.73	1.49
Ramachandran (analyzed/outliers)	420/13	871/5

[a] $R_{sym} = \Sigma|I_{avg}-I_i|/\Sigma I_i$

[b] R factor = $\Sigma|F_p - F_{pcalc}|/\Sigma F_p$, where F_p and F_{pcalc} are the observed and calculated structure factors; R_{free} is calculated with 5% of the data.

Table S1. Crystallographic parameters, data collection and refinement statistics.

	$G6PD^{K403QdLtL}$ tetramer	$G6PD^{K403QdLtL}$ dimer
**Data collection parameters**		
Instrument	SSRL BL4-2	SSRL BL4-2
Type of Experiment	SEC-SAXS	SEC-SAXS
Defining slits size (H mm × V mm)	0.15 × 0.15	0.15 × 0.15
Detector distance (m)	2.5	2.5
Detector	Pilatus3 X 1M	Pilatus3 X 1M
Beam energy (keV)	12.0	12.0
q range ($Å^{-1}$)	0.0050–0.389	0.0050–0.389
Sample cell	Quartz capillary (D=~1.2mm)	Quartz capillary (D=~1.2mm)
Temperature (K)	298	298
Exposure time/frame (s)	1	1
Frames per SEC-SAXS data set	500	500
Number of blank images used for averaging	100	100
Number of sample images used for averaging	5	5
Image numbers used for averaging	305-314	335-349
SEC column	Superdex 200 Increase PC 3.2/300	Superdex 200 Increase PC 3.2/300
HPLC flow rate (mL/min)	0.05	0.05
Sample concentration (mg/ml)	10	10
SEC injection volume (µL)	30	30
Buffer	50 mM Tris-HCl (pH 7.4), 150 mM NaCl	50 mM Tris-HCl (pH 7.4), 150 mM NaCl
**Software employed**		
Primary data reduction	*SasTool/SECPipe*	*SasTool/SECPipe*
Data processing	*PRIMUS*	*PRIMUS*
P(r) analysis	*GNOM*	*GNOM*
Atomistic modeling	*CORAL*	*CORAL*
**Structural parameters**		
Guinier analysis		
$I(0)$ (cm^{-1})	0.20 ± 0.00035	0.048 ± 0.00016
R_g (Å)	44.16 ± 0.11	38.23 ± 0.19
q_{min} ($Å^{-1}$)	0.012	0.012
qR_g range	0.35 - 1.30	0.36 - 1.29
P(r) analysis		
$I(0)$	0.20	0.05
R_g (Å)	44.25	38.53
D_{max} (Å)	147.99	127.00
q range ($Å^{-1}$)	0.0095 – 0.211	0.0095 – 0.21
Porod volume estimate ($Å^3$)	371000	179000
**Atomistic Modeling (CORAL)**		
q range ($Å^{-1}$)	0.0050 – 0.30	0.0066 – 0.30
Number of repetitions	20	20
χ^2 range	1.28 – 1.30	1.27 – 1.33
Predicted R_g of the best model (Å)	43.05	37.47
Predicted D_{max} of the best model (Å)	152	133.0

Table S2. SAXS data collection and analysis of the $G6PD^{K403QdLtL}$ tetramer and dimer.

Movie S1-S6 figure legends

Movie S1. Structural morphing of the G6PD dimer. The G6PD^{WT} (PDB 6E08) dimer morphed into G6PD^{K403Q} (PDB 7SEI), white to salmon respectively, followed by G6PD^{K403Q} (PDB 7SEI) morphed into G6PD^{K403QdLtL} (PDB 7SEH) dimer, salmon to teal.

Movie S2. Structural morphing of the G6PD dimer rotated 90°. The G6PD^{WT} (PDB 6E08) dimer morphed into G6PD^{K403Q} (PDB 7SEI), white to salmon respectively, followed by G6PD^{K403Q} (PDB 7SEI) morphed into G6PD^{K403QdLtL} (PDB 7SEH) dimer, salmon to teal.

Movie S3. Structural morphing of the αf-helix. The G6PD^{WT} (PDB 2BHL) αf-helix morphed into the G6PD^{K403Q} (PDB 7SEI) αf-helices, white to salmon respectively, with the G6P (2BHL) and catalytic NADP$^+$ (7SEI) displayed.

Movie S4. Structural morphing of the G6PD tetramer. The G6PD^{WT} (PDB 6E08) tetramer morphed into the G6PD^{K403Q} (PDB 7SEI) tetramer, white to salmon respectively.

Movie S5. Structural morphing of opposing αj-helices at the tetramer interface. The G6PD^{WT} (PDB 6E08) αj-helices morphed into the G6PD^{K403Q} (PDB 7SEI) αj-helices, white to salmon respectively.

Movie S6. Structural morphing of the αj-helix of one monomer and structural NADP$^+$ ß-sheet from a second monomer across the tetramer interface. The G6PD^{WT} (PDB 6E08) αj-helix and structural NADP$^+$ ß-sheet morphed into the G6PD^{K403Q} (PDB 7SEI) αj-helix and structural NADP$^+$ ß-sheet, white to salmon respectively.

Experimental procedures

Quantification of SEC chromatogram

The chromatogram was deconvoluted into discrete peaks with Igor Pro multipeak fitting 2 package. To generate a fit, the lowest possible number of peaks were used, followed by a subsequent addition of peaks until the chromatogram was well represented. One chromatogram was analyzed for each condition.

Morph Videos

Using Pymol software, the G6PD^{WT} (PDB 6E08) monomer was used to generate a tetramer structure by combining three symmetry equivalent monomers. A hypothetical G6PD^{K403Q} tetramer structure was generated from the biological dimer structure of G6PD^{K403Q} mutant (PDB 7SEI) by aligning the G6PD^{K403Q} dimer to each dimer of the G6PD^{WT} tetramer. The three tetramer structures were then used to generate morphing videos, using Chimera's morph feature.
